# Mechanistically Different Mechanochromophores Enable Calibration and Validation of Molecular Forces in Glassy Polymers and Elastomeric Networks

**DOI:** 10.1002/anie.202409369

**Published:** 2024-10-17

**Authors:** Raphael Hertel, Maximilian Raisch, Michael Walter, Günter Reiter, Michael Sommer

**Affiliations:** ^1^ Department of Polymer Chemistry Institute for Chemistry Chemnitz University of Technology Str. der Nationen 62 09111 Chemnitz Germany; ^2^ FIT Freiburg Center for Interactive Materials and Bioinspired Technologies Albert-Ludwig-University of Freiburg Georges-Köhler-Allee 105 79110 Freiburg Germany; ^3^ Institute of Physics Albert-Ludwig-University of Freiburg Hermann-Herder-Str. 3 79104 Freiburg Germany

**Keywords:** Polymers, Mechanical properties, Mechanochromism, Chromophores, Molecular force probes

## Abstract

Sterically distorted donor‐acceptor π‐systems, termed DA springs, can be progressively planarized under mechanical load causing a bathochromic shift of the photoluminescence (PL) spectrum. By combining theory and experiment, we here use a simple linear force calibration for two different conformational mechanochromophores to determine molecular forces in polymers from the mechanochromic shift in PL wavelength during multiple uniaxial tensile tests. Two systems are used, i) a highly entangled linear glassy polyphenylene and ii) a covalent elastomeric polydimethylsiloxane network. The mean forces estimated by this method are validated using known threshold forces for the mechanochemical ring‐opening reactions of two different spiropyran force probes. The agreement between both approaches underlines that these DA springs provide the unique opportunity for the online monitoring of local molecular forces present in diverse polymer matrices.

## Graphical abstract

Polymers with covalently incorporated donor‐acceptor torsional springs allow for real‐time monitoring of forces, that are calibrated using known spiropyran mechanophores. This concept is demonstrated using glassy, entangled polymers and elastomeric networks.

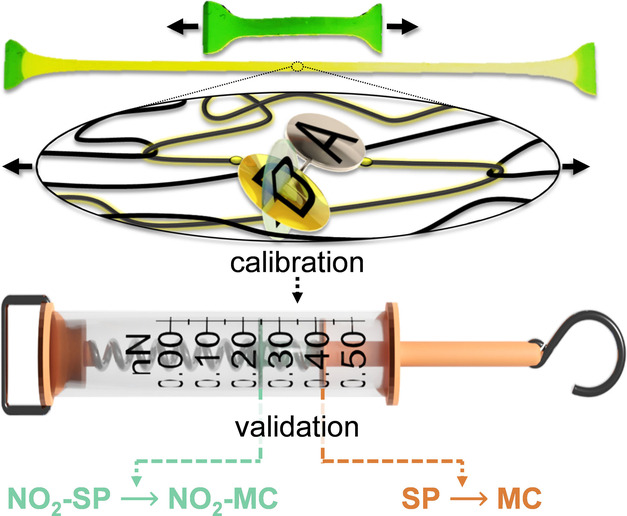

The ability to capture and map the stressed state of polymer materials using optical stress reporters at the molecular level addresses a number of fundamental questions of technological importance. For example, stability and longevity of polymer networks and composites are governed by homogeneity, stress dissipation and stress distribution.[^1^, ^2^, ^3^, ^4^] Furthermore, mechanically functional polymers may respond to stress by completely changing polymer properties^[5]^ and polymer architecture; e.g. force‐generated secondary chemical reactions may enable mechanical reinforcement.[^6^, ^7^] In biology‐related areas, efforts were made to measure mechanical forces at the molecular level.[^8^, ^9^, ^10^]

A multitude of chromophores that allow and control such changes, termed mechanochromophores,^[11]^ has been presented.[^12^, ^13^, ^14^, ^15^, ^16^, ^17^, ^18^, ^19^] Most of these function through mechanochemical reactions,[^3^, ^20^, ^21^, ^22^, ^23^] changes in noncovalent interactions,[^24^, ^25^, ^26^] structural changes such as conformation[^27^, ^28^, ^29^] and configuration^[30]^ or combinations of these factors.[^31^, ^32^, ^33^, ^34^, ^35^, ^36^]

While the majority of studies has so far focused on qualitative proof‐of‐concepts of new mechanochromophores, a quantification of involved forces determined from the corresponding mechanochromic response is often tedious and limited to a few examples. Studies that measure or map mechanical stress by virtue of mechanochromophores usually require an external calibration, either by correlating stress values derived from mechanical tests or simulated stress.[^1^, ^2^, ^37^, ^38^] Using the stress of a macroscopic sample as a function of deformation (here referred to as macroscopic stress) for calibration, molecular forces acting on single polymer chains can be estimated if the chain or crosslink density is known.^[39]^ Experimentally, molecular forces have been determined in polydimethylsiloxane (PDMS) by kinetic studies of mechanophore reactions at different strains[^40^, ^41^] using the Eyring‐Bell‐Evans model.[^42^, ^43^, ^44^] In addition to tedious real‐time force analysis, the fraction of polymer strands probed and forces acting on the other strands are not definite. Consequently, the quantified forces largely differ in their magnitude.[^40^, ^41^] On the theoretical side, very few multiscale simulations addressing force transduction from bulk to single chains during mechanophore activation have emerged.[^45^, ^46^]

Previously, we have presented distorted donor‐acceptor (DA) π‐systems, termed DA torsional springs, as conformational mechanophores and have demonstrated their unique ability to monitor stress in bulk specimens online and reversibly.[^47^, ^48^, ^49^] Here, we use DA springs to quantify molecular forces acting locally on glassy and entangled polymers as well as covalent elastomeric networks. Force‐induced progressive planarization of the π‐system causes a bathochromic shift of the photoluminescence (PL) spectrum ▵*λ*_exp_, the magnitude of which depends on the stress that builds up in the material. ▵*λ*_exp_ is compared to the simulated force‐dependent shift of the charge transfer (CT) wavelength ▵*λ*_sim_. This relation is finally validated using spiropyran‐based mechanochromophores, for which critical forces required for the spiropyran‐merocyanine (SP‐MC) isomerization are known. Figure 1 depicts this approach schematically.

**Figure 1:**
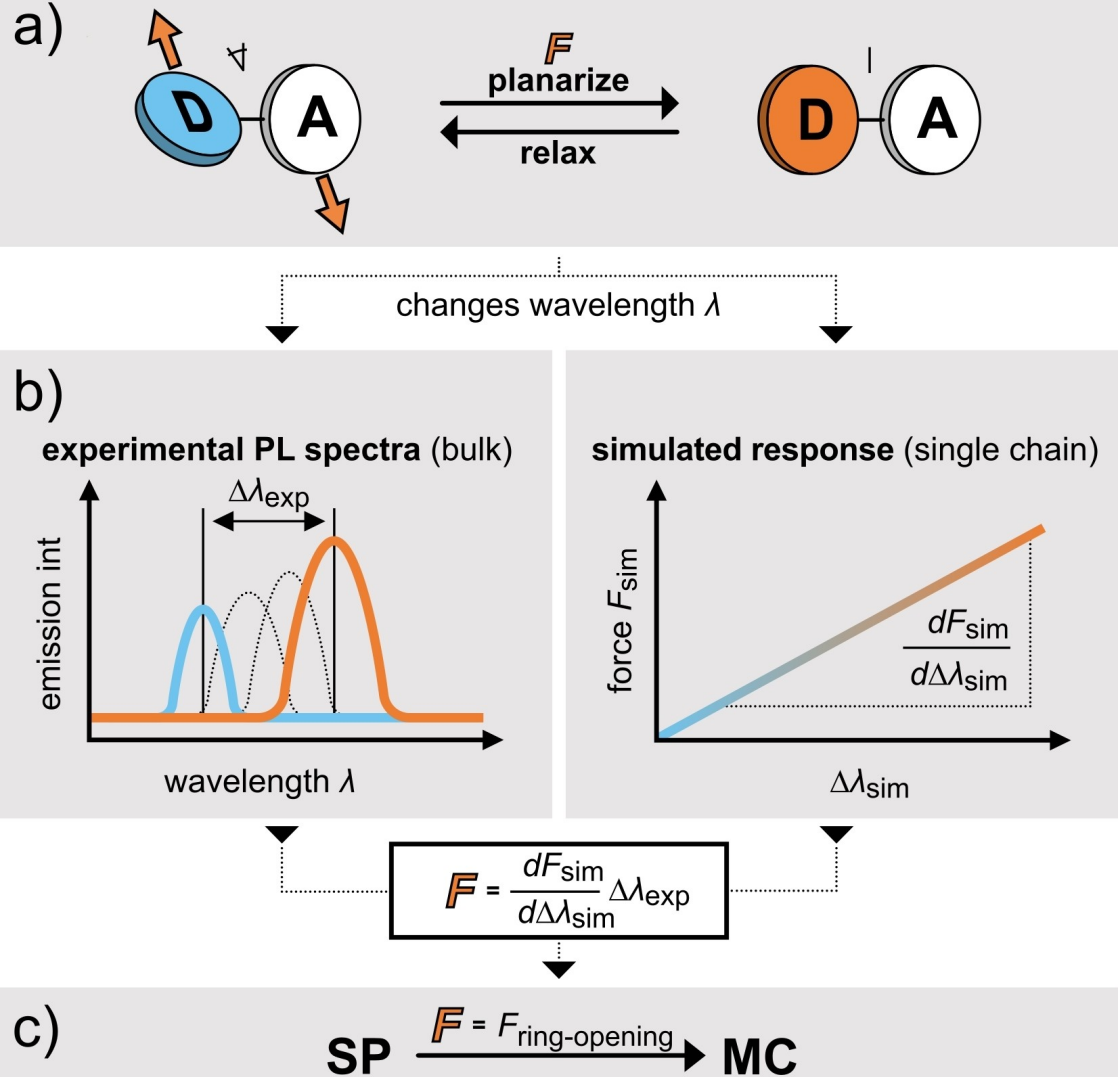
Approach to calibrate and validate molecular forces in bulk materials probed by DA conformational mechanochromophores. a) Schematic structure of the DA force probes. Note that while the schematic structure is DA for simplicity, experimentally symmetric DAD structures are used. b) Calculation of the molecular forces *F* from experimental bulk data and simulated single chain forces. c) Validation of the obtained forces using known SP mechanophores.

We first measured ▵*λ*_exp_ of a number of samples containing diphenyldiketopyrrolopyrrole (**DPP**)^[47]^ and dithienylquinoxaline (**TQxT**)^[49]^ conformational springs covalently incorporated into poly(*meta,meta*,*para*)phenylene (P*mmp*P) (Scheme 1). ▵*λ*_sim_ was simulated using the constrained geometries simulate external force (CoGEF) method^[50]^ and time‐dependent density‐functional theory (TD‐DFT). CoGEF is equivalent to isotensional methods^[51]^ for continuous deformations. Assuming that the Stokes shift is independent of deformation, we equate ▵*λ*_sim_ with ▵*λ*_exp_. For **DPP**, ▵*λ*_sim_ is proportional to the force *F* applied on a single chain for *F*≤1 nN with a proportionality factor of 17±2 nm nN^−1^.^[47]^ ▵*λ*_exp_ of **TQxT** is determined by two contributions: at small forces, conformational rearrangement occurs that involves the flipping of the aliphatic bridge and thus thiophene orientation, followed by force‐induced planarization at larger forces.^[49]^ In order to isolate the contribution from **TQxT** planarization that is also proportional to forces≤1 nN with a proportionality factor of 21±3 nm nN^−1^,^[49]^ the PL shift arising from the ring flip ▵*λ*_ring flip_ was subtracted from the total shift. Thus, molecular forces *F* can be determined in materials directly without external calibration (given the forces are in the linear region) from ▵*λ*_em_ for **DPP** using

(1) $$F=\frac{\Delta \lambda_{em,DPP}}{17\;\;nm\;nN^{-1}}$$

**Scheme 1:**
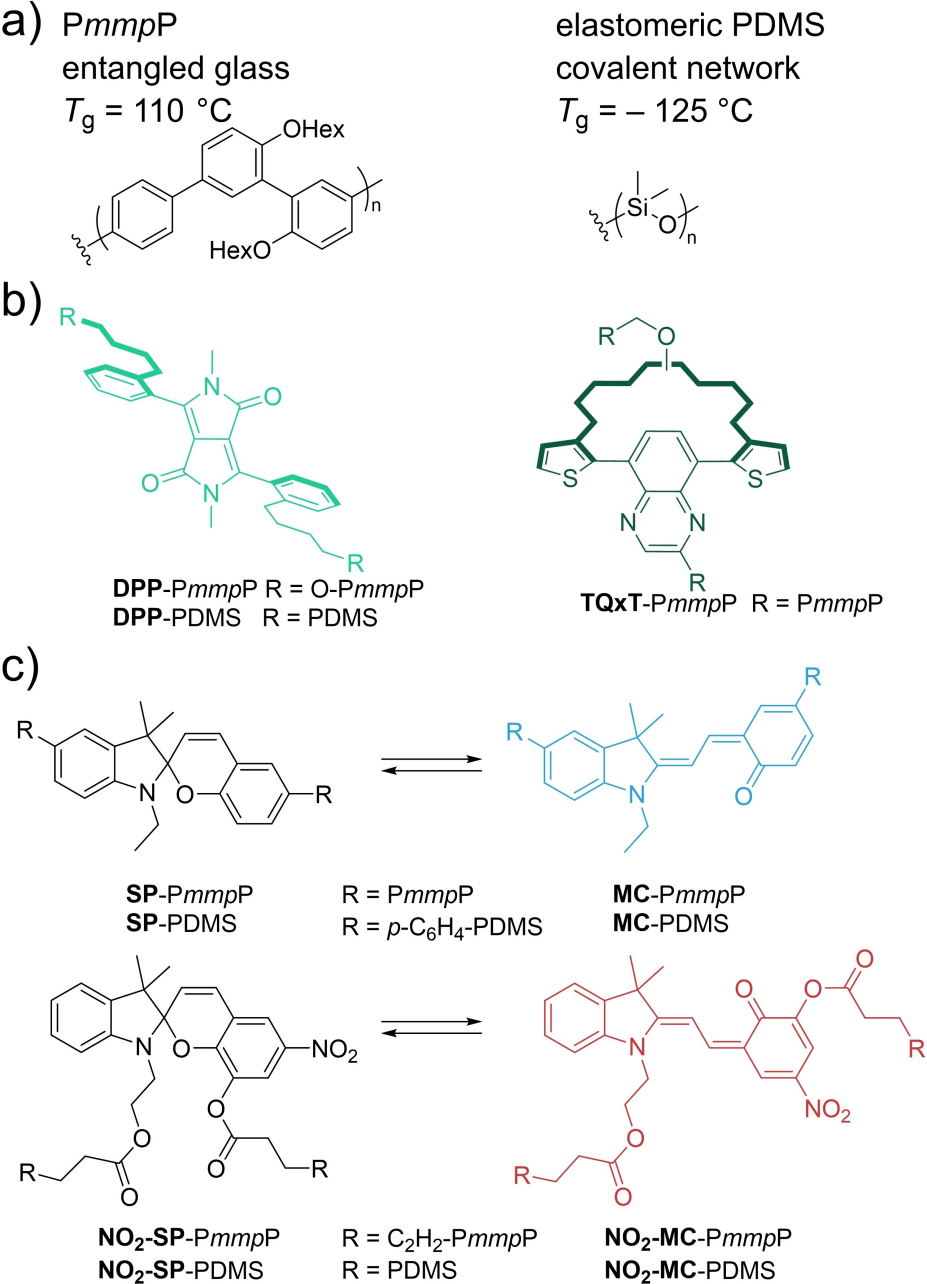
Chemical structures of all mechanochromophores and covalent polymer matrices used: a) glassy P*mmp*P and elastomeric PDMS, b) conformational mechanochromophores based on diphenyldiketopyrrolopyrrole **DPP** and dithienylquinoxaline **TQxT**, as well as c) two SP probes and their MC forms.

and for **TQxT** using

(2) $$F=\frac{\Delta \lambda_{em,TQxT}-\Delta \lambda_{ring\;flip}}{21\;\;nm\;nN^{-1}}$$

Applying calibrations (1) and (2) we evaluated the optical response of a large number of specimens prepared from **DPP**‐ and **TQxT**‐functionalized P*mmp*P. Sufficient entanglements for mechanical stability and an efficient force transduction were ensured by using polymer samples with large molecular weights (*M*_n,SEC_>25 kg mol^−1^, *M*_w,SEC_≥100 kg mol^−1^) that are well beyond the entanglement molecular weight *M*_e_=4.8 kg/mol.[^48^, ^52^] Films were drop cast (see SI) and cut for uniaxial tensile testing. The molecular forces determined by **DPP** or **TQxT** springs in P*mmp*P not only show the same qualitative trend, but also coincide, although both systems differ in chemical structure, their mode of linkage and mechanochromophore concentration. This observation indicates the validity of the linear calibration of both conformational mechanophores under the experimental conditions.

Since the stress‐strain experiments with glassy P*mmp*P exhibited good reproducibility, mean molecular forces could be obtained from multiple tensile tests as a function of the strain *ϵ* (Figure 2a, b). A linear dependence between mean molecular force and macroscopic stress is seen at *ϵ* >50 % (the scattered data at *ϵ* <50 % are caused by neck formation and propagation into the focus of detection). The Pearson correlation coefficient between macroscopic stress and molecular force amounts to 0.998 (Figure S3). This is in accordance with rheological measurements, where the macroscopic shear stress in glassy PMMA could be composed of all locally acting stresses derived from mechanophore activation.^[53]^ In the plastic deformation regime, the forces acting on the DA springs during further neck propagation are rather constant at 0.16±0.03 nN with a mean macroscopic engineering stress of 30±2 MPa. In the subsequent strain hardening regime at *ϵ* >140 %, both the macroscopic engineering stress and the mean molecular force increase linearly with *ϵ*. This underlines the experimental linear dependence between ▵*λ*_exp_ and the applied force. Due to the broadness of the emission spectra multiple force populations could be underlying. Specimen failure occurs at *ϵ*=270 %, a macroscopic engineering stress of 50±3 MPa and a mean estimated maximal molecular force of 0.46±0.06 nN. We note that these force values are average values and there are likely repeat units experiencing higher or lower forces.[^54^, ^55^]

**Figure 2:**
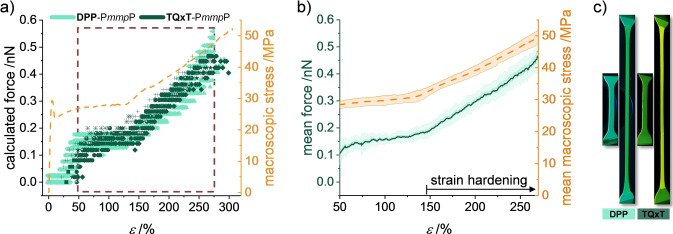
a) Molecular force obtained from ▵*λ*_exp_ and ▵*λ*_sim_ for multiple tensile tests of **DPP**‐P*mmp*P (8 measurements) and **TQxT**‐P*mmp*P (10 measurements), as well as a representative stress‐strain curve (dashed yellow line). b) Mean force (solid green line) ± standard deviation (green area) acting on the P*mmp*P chains from Figure 2a and mean macroscopic stress (dashed yellow line) ± standard deviation (yellow area) of the tensile tests. c) Photographs of **DPP**‐P*mmp*P and **TQxT**‐P*mmp*P samples before and after deformation.

The molecular forces estimated by this approach appear to be larger but of the same order of magnitude compared to 0.15 nN, which is the force per chain obtained from the true macroscopic stress and the mean cross‐sectional area of a repeat unit (see Supporting Information for details). However, the cross‐sectional area of a repeat unit approximated by this approach equals the area per polymer chain perpendicular to the tensile axis under the assumption that all chains are completely aligned with the direction of strain. Due to the bulky and rigid nature of the repeat units and quasi‐random chain directions arising from their amorphous nature, a parallel arrangement of the chains is unlikely leading to larger effective polymer chain cross‐sectional areas perpendicular to the strain direction and thus larger forces per polymer chain. The higher mean molecular forces might additionally be attributed to an underestimated true stress that was calculated without considering the elastic reduction in sample diameter. As the entanglements are highly important for force transduction,^[48]^ we also note that the estimated force per entanglement is 0.9 nN (see SI). This may present a more suitable description of the observed forces.

To further demonstrate validity of this approach, we compared the mechanochromic behavior of **DPP** and **TQxT**‐functionalized P*mmp*P to analogous samples with two SP force probes known to undergo ring‐opening at forces between 0.2 to 0.4 nN,[^56^, ^57^, ^58^] which is the relevant force range of the strain hardening regime of P*mmp*P. Covalent incorporation of two different SPs (see Supporting Information for details of synthesis and characterization) furnished **SP**‐P*mmp*P and **NO_2_‐SP**‐P*mmp*P and their MC isomers **MC**‐P*mmp*P and **NO_2_‐MC**‐P*mmp*P, respectively (Scheme 1c). Forces required for this isomerization are substituent‐dependent,[^56^, ^57^] and cause changes in the UV/Vis spectra when the molecular forces are above a certain threshold value. For **SP**‐P*mmp*P, ring‐opening occurs at ~0.4 nN at loading rates between 10^−5^ and 10^−1^ nN s^−1^ from 3S‐CoGEF calculations considering the force dependent barriers overcome by temperature.^[56]^ NO_2_‐substitution of SP in 6‐position is known to stabilize the MC form, and lowers the barrier for the force‐induced reaction.[^57^, ^59^] Taking the effect of finite temperature into account reveals forces that are in line with results from single molecule force spectroscopy (SMFS), where *para*‐NO_2_‐substituted SPs were reported to ring‐open already at forces as low as 0.24 nN.^[58]^ For the P*mmp*P matrix, based on the preceding observations (Figure 2), we anticipate that some chains experience forces above these threshold values in the plastic deformation and the strain hardening regime for **NO_2_‐SP** and **SP**, respectively. Thus, **SP** and **NO_2_‐SP** are most ideal force probes for the validation of the presumed force range.

Deformation‐dependent relative changes in UV/Vis absorption of **MC**‐P*mmp*P (*λ*=650 nm) and **NO_2_‐MC**‐P*mmp*P (*λ*=630 nm) were monitored in situ in transmission (Figure 3, for experimental details, full spectra, and data analysis, see SI).

**Figure 3:**
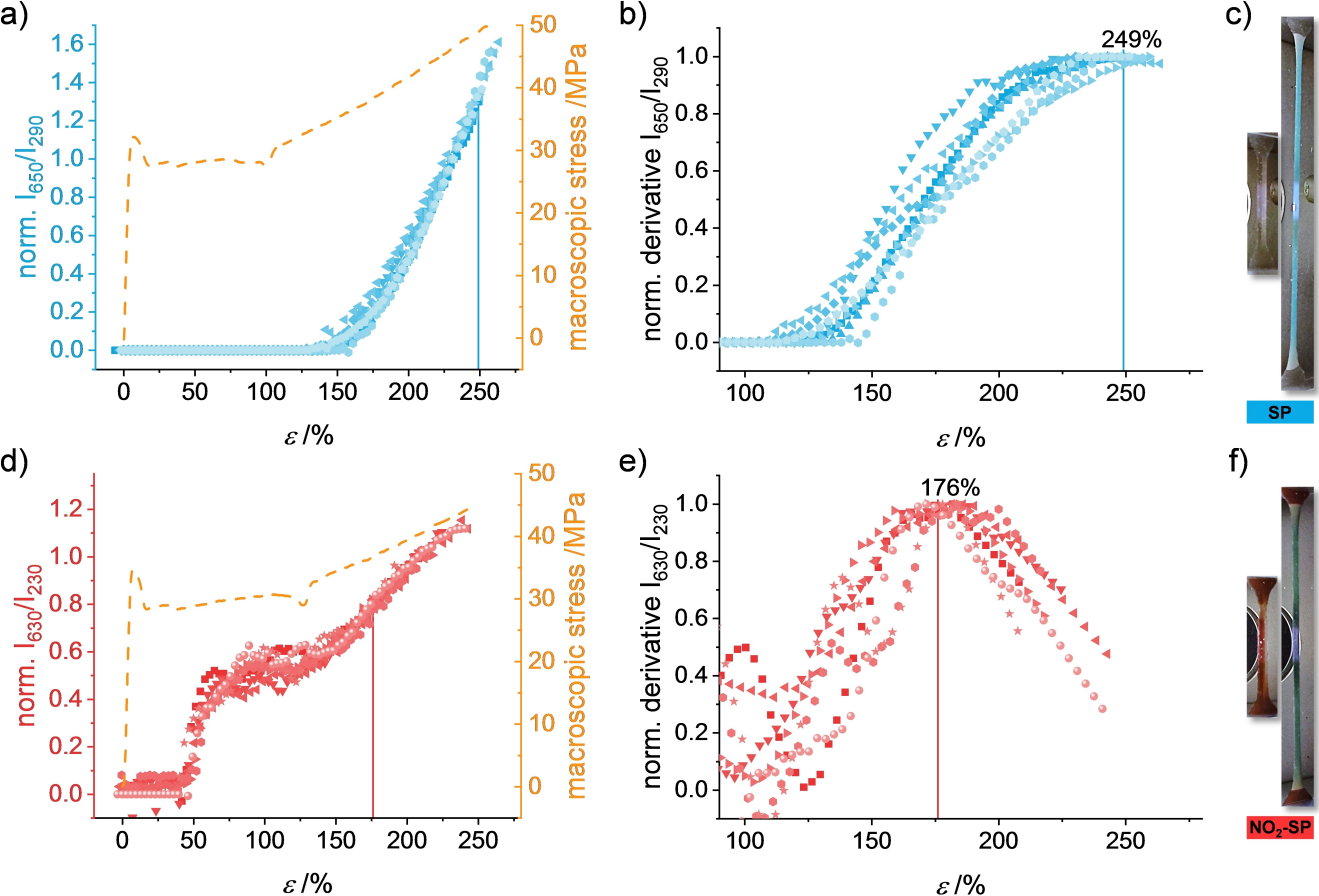
The UV/Vis absorbance of MCs *I*_650_ and *I*_630_ relative to the absorbance of the matrix *I*_290_ and *I*_230_ normalized to the value at *ϵ*=225 % and 205 % (left) with representative stress‐strain curves (dashed yellow lines) and first derivatives of the relative UV/Vis absorbance (middle) as a function of *ϵ* from **MC**‐P*mmp*P (a,b) and **NO_2_‐MC**‐P*mmp*P (d,e), respectively. The subscript numbers are the wavelengths of the respective absorption bands. Right: photographs of films from **SP**‐P*mmp*P (c) and **NO_2_‐SP**‐P*mmp*P (f) before and after deformation.

SMFS experiments revealed that the forces required for the SP‐MC isomerization decrease with increasing matrix polarity.^[60]^ Due to the negative solvatochromism of MC^[61]^ we anticipate a low matrix polarity from the redshifted absorption maximum of **NO_2_‐MC**‐P*mmp*P throughout the entire process of deformation. Therefore, the reduction in the threshold forces for the ring‐opening reaction of **SP** and **NO_2_‐SP** due to matrix polarity should be negligible.

To account for specimen thinning during straining, the MC absorbance intensities *I*_650_ and *I*_630_ were analyzed relative to the matrix absorbance intensities *I*_290_ and *I*_230_. The forces acting on single polymer chains first exceed the threshold force for **SP**‐P*mmp*P ring‐opening at *ϵ* >130 % close to the onset of strain hardening (Figure 3a). On the contrary, the threshold force for the mechanochromic reaction of **NO_2_‐SP**‐P*mmp*P is already reached at *ϵ* ≈50 % by a fraction of polymer chains as soon as the neck enters the detected region (Figure 3d). The local molecular force monitored with **NO_2_‐SP**‐P*mmp*P seems to be constant in the plastic deformation regime, as the relative absorbance does not increase further until strain hardening. This agrees with the observed ▵*λ*_exp_ of the conformational mechanochromophores **DPP**‐P*mmp*P and **TQxT**‐P*mmp*P. Unlike **MC**‐P*mmp*P, the relative absorbance of **NO_2_‐MC**‐P*mmp*P approaches a constant value before sample failure. These trends were analyzed with respect to changes during deformation by inspecting the first derivatives, as shown in Figures 3b and 3e. Since deformation increased at a constant strain rate, we expect the first derivatives to match the trend of the reaction rate of SP‐MC conversion at the respective strain values. While the derivative of the ratio *I*_650_/*I*_290_ of **MC**‐P*mmp*P appears to further increase beyond *ϵ*=230 %, the same analysis delivers a maximum at *ϵ*=176 % for **NO_2_‐MC**‐P*mmp*P. We assume that at a given deformation, the actual force acting on the SP derivatives is distributed.[^54^, ^55^] Therefore, and because of the stochastic nature of the force‐induced SP‐MC transitions,[^62^, ^63^] even at small *ϵ* some of the SP mechanophores already experience a force high enough for isomerization. After the mean molecular force reaches the maximum of the force distribution for SP‐MC isomerization, progressively fewer SPs will additionally isomerize. In other words, the force necessary for the ring‐opening reaction is given by the mean of the force distribution at the strain *ϵ* featuring the highest SP‐MC reaction rate, which in turn is obtained from the maximum of the first derivative of the relative absorbance.

Since the mean molecular force obtained from the DA springs increases linearly with *ϵ* in the strain hardening regime (cf. Figure 2b), such correlation is straightforward for *ϵ* >140 %. In Figure 4, the forces for the activation of **NO_2_‐SP**‐P*mmp*P and **SP**‐P*mmp*P are estimated to be 0.25±0.03 nN and≥0.40 nN, respectively. These values are in good agreement to known values for both 3S‐CoGEF calculations[^56^, ^62^, ^63^] which are very similar to isotensional approaches^[51]^ as well as SMFS experiments,[^58^, ^64^] confirming that the herein obtained values determined by DA springs are reasonably good descriptors of forces acting on polymer chains.

**Figure 4:**
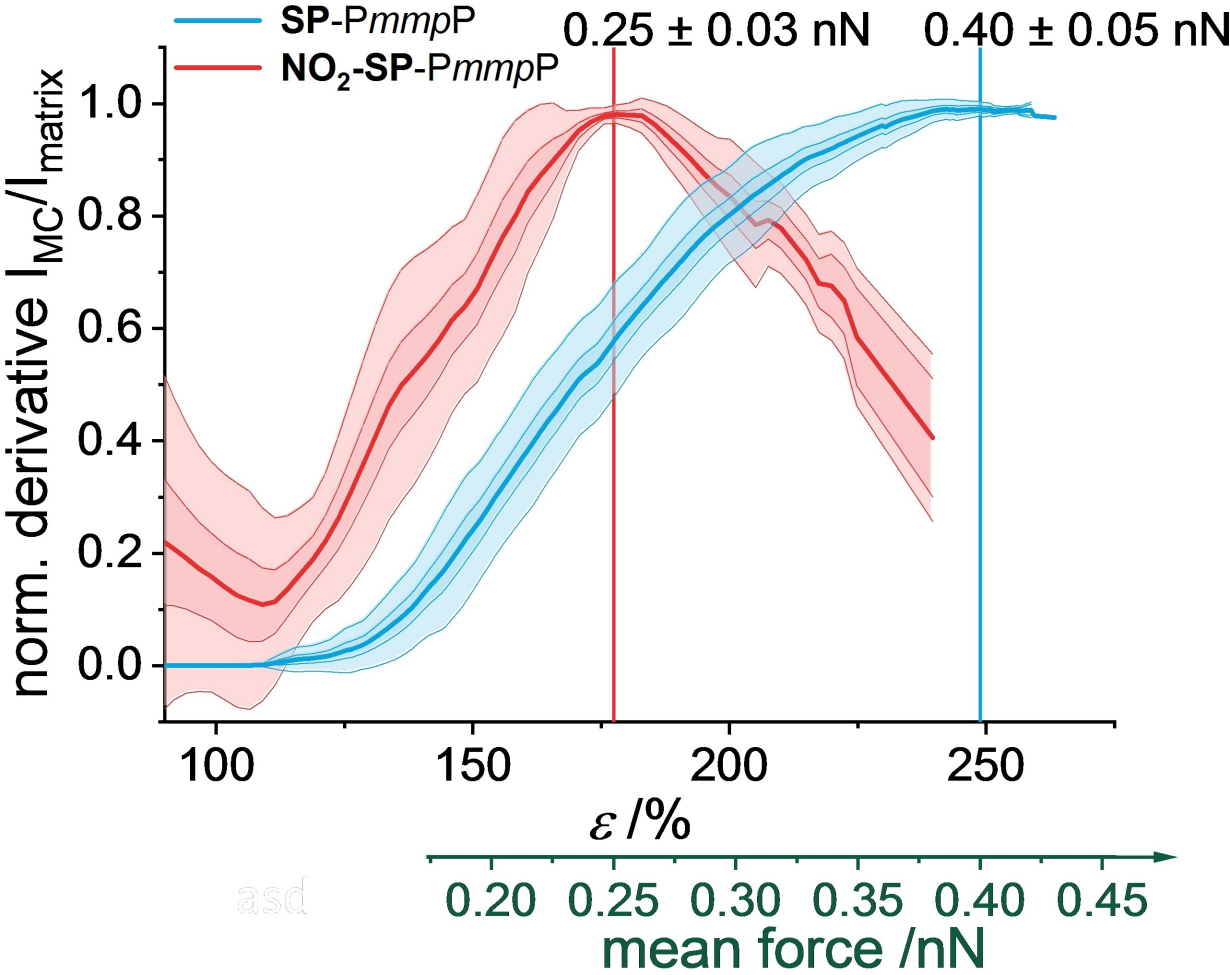
Mean first derivative (thick line) of the MC UV/Vis absorbance ± standard deviation (colored area) of **MC**‐P*mmp*P (blue) and **NO_2_‐MC**‐P*mmp*P (red) relative to the matrix absorbance *I*_MC_/*I*_matrix_ as a function of *ϵ* and mean molecular force determined from Figure 2b.

P*mmp*P is an amorphous, highly ductile polymer with a *T*_g_ of 110 °C and mechanical behavior similar to aromatic polycarbonate.^[56]^ This situation may not be representative for other polymer systems involving lower *T*_g_s or semicrystalline morphologies. In order to further test the herein developed approach and prove that the working principle of DA springs is not limited to glassy polyphenylenes, divinyl‐functionalized **DPP** was covalently incorporated into PDMS (Sylgard 184, see Scheme S2). To compensate for the lower stress and strain window of PDMS, the elastomer samples were cut to obtain an unconventional shape that allows to concentrate the stress and strain in the center of the specimen where detection takes place (see inset of Figure 5). Applying the same force calibration as for P*mmp*P, the molecular forces were calculated from ▵*λ*_exp_ as a function of *ϵ* for multiple tensile tests (Figures 5 and S6). The onset of the mechanochromic response is located at *ϵ* >100 % just before the strain hardening. The determined mean molecular force is again a linear function of the mean macroscopic stress for *ϵ* >120 % in PDMS (Figure S7). A force of 0.03 nN is measured at an engineering strain of *ϵ*=130 % and 1.2 MPa macroscopic stress in our experiments with the **DPP**‐PDMS force probe, which marks the lower detection limit of the **DPP** spring. Due to the specimen shape, the local true strain is largest in the center of the specimen. Thus, the determined 0.03 nN can be seen as an upper limit along the tensile axis. Molecular forces between 0.004 nN^[41]^ and 0.03 nN^[40]^ per PDMS strand have been determined by kinetic studies on azobenzene and SP mechanophores, respectively, at similar stress and strain values. Our results align with this force range, confirming the usefulness of the presented approach. At strain at break, the mean molecular force in the center of the specimen obtained with **DPP**‐PDMS is 0.17±0.02 nN.

**Figure 5:**
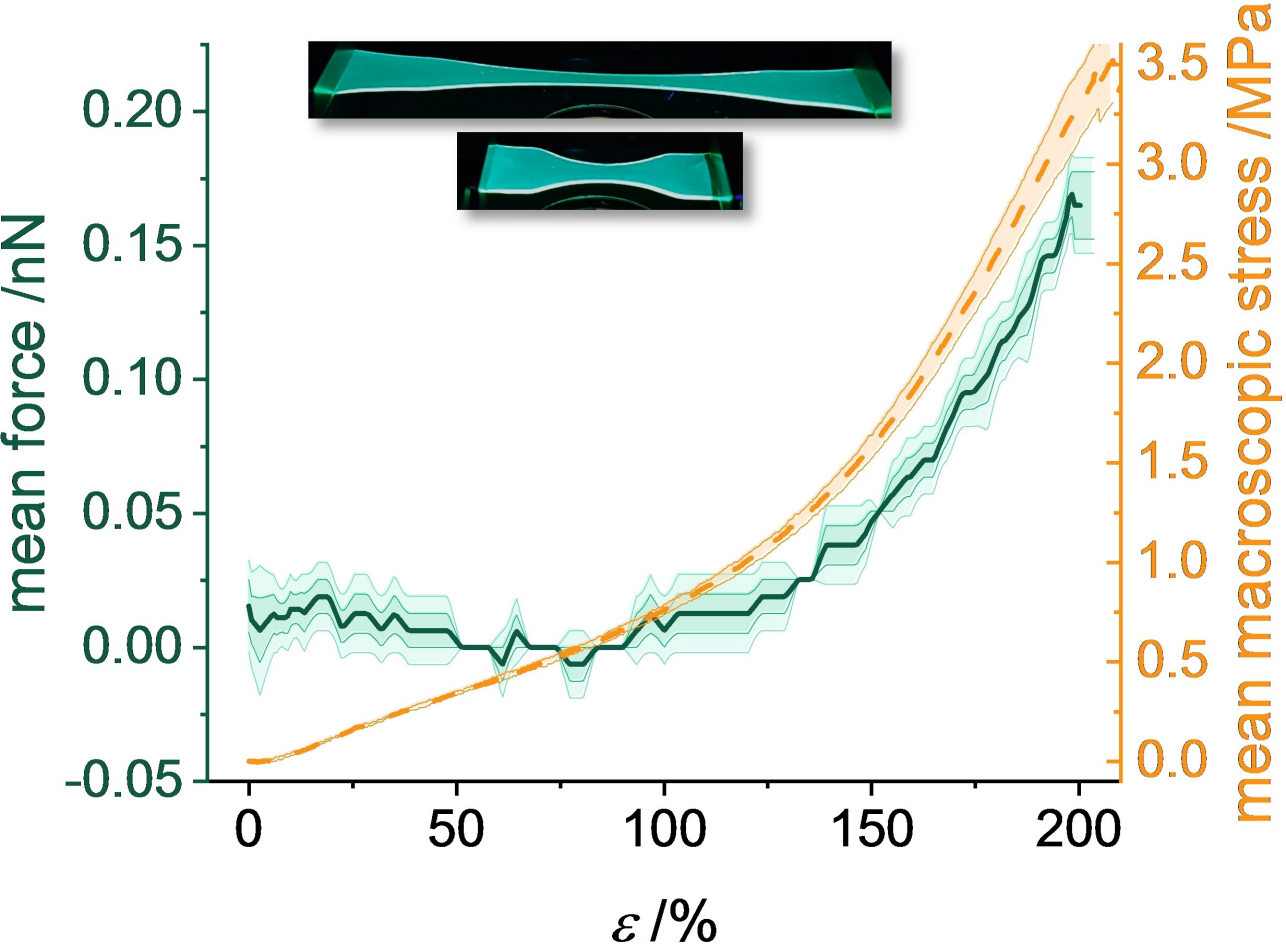
Mean force (solid green line) ± standard deviation (green area) on **DPP**‐PDMS springs calculated from ▵*λ*_exp_ during tensile testing and mean macroscopic stress (dashed yellow line) ± standard deviation (yellow area) as functions of *ϵ*. Inset: photographs of the **DPP**‐PDMS samples at *ϵ*=0 % and 200 %.

To verify the magnitude of force values obtained from **DPP**‐PDMS, divinyl‐functionalized **SP** and **NO_2_‐SP** were prepared and incorporated into PDMS (Scheme S3). Again, the relative *ϵ*‐dependent absorbance of the MC forms was monitored (Figures S8 and S9). The activation of **NO_2_‐SP**‐PDMS is visible at *ϵ* >70 %, whereas **SP**‐PDMS exhibits the onset of the force‐induced ring‐opening reaction at *ϵ*>120 %. In both cases, a maximum cannot be reached when plotting the *ϵ*‐dependent first derivatives of the relative absorbance (Figure S9). We therefore expect the mean molecular force to be smaller than 0.24 nN in the entire strain range probed, but large enough for some strands to exceed the threshold of 0.4 nN for **SP** activation, despite stochastic SP‐MC transitions at finite temperature at lower forces. The absorption bands of **MC**‐PDMS (*λ*=630 nm) and **NO_2_‐MC**‐PDMS (*λ*=600 nm) are hypsochromically shifted compared to the respective P*mmp*P polymers. However, regarding the large negative solvatochromism of MC^[61]^ and the low Hildebrandt solubility parameter of PDMS,^[65]^ the low polarity of the PDMS matrix is not expected to reduce the threshold force for the ring‐opening reaction.^[60]^ The force at which a siloxane bond most likely ruptures in PDMS has been determined to be 1.3 nN by SMFS.^[66]^ Assuming a potentially multimodal or exponential distribution of the acting molecular forces,^[67]^ e. g. considering non‐uniform strand lengths,^[68]^ a small quantity of polymer strands may exceed that force at strain at break. The determined maximal mean molecular force from **DPP**‐PDMS at break of 0.17±0.02 nN is in line with the observed mechanochemistry from both **NO_2_‐SP**‐PDMS and **SP**‐PDMS taking the broader force distribution in PDMS into account. Nevertheless, due to the soft nature of PDMS, the force range represents a lower limit for quantification according to the approach used herein. Further studies are needed to provide in‐depth insight in systems with complex force distributions.

We have presented an approach to quantify molecular forces in polymer systems using different conformational mechanochromophores, termed donor‐acceptor (DA) springs. Molecular forces could be obtained from the correlation of the force‐induced shift of photoluminescence spectra ▵*λ*_em_ and the simulated force‐dependent shift of the charge‐transfer wavelength ▵*λ*_sim_. By averaging the results of multiple uniaxial tensile tests, we determined the mean molecular forces during straining of an entangled glassy polyphenylene and compared them to the theoretical force per chain estimated from the repeat‐unit cross‐sectional area and the macroscopic stress. The determined forces were experimentally validated using two different spiropyran force probes. Additionally, the approach was extended to elastomeric polydimethylsiloxane(PDMS) demonstrating broader scope and applicability. Again, the mean molecular forces during uniaxial tensile testing were determined using the same calibration as for the entangled glassy polymer and likewise cross‐checked against well‐known spiropyran mechanochemistry. Remarkably, for both entangled, glassy polyphenylene as well as for elastomeric PDMS, the determined mean molecular forces were linear functions of the measured macroscopic stress. Overall, the results approve DA conformational springs as excellent force probes also for other polymer materials without the necessity for prior calibration, provided that the forces are in the linear response regime of the DA springs. Even though the forces from single molecule simulations and polymer tensile testing differ by 10 orders of magnitude, this method enables the correlation between molecular forces and ▵*λ*_em_.

## Supporting Information

The authors have cited additional references within the Supporting Information.[^69^, ^70^, ^71^, ^72^, ^73^]

## Conflict of Interests

The authors declare no conflict of interest.

## Supporting information

As a service to our authors and readers, this journal provides supporting information supplied by the authors. Such materials are peer reviewed and may be re‐organized for online delivery, but are not copy‐edited or typeset. Technical support issues arising from supporting information (other than missing files) should be addressed to the authors.

Supporting Information

## Data Availability

The data that support the findings of this study are available from the corresponding author upon reasonable request.
