# Supplementary material for: Mechanistically Different Mechanochromophores Enable Calibration and Validation of Molecular Forces in Glassy Polymers and Elastomeric Networks
Source: Angew Chem Int Ed Engl. 2024 Oct 17;63(49):e202409369. doi: 10.1002/anie.202409369 (PMC11586691; doi:10.1002/anie.202409369)
Supplement: Supplementary file 1 — Supporting Information [file ANIE-63-e202409369-s001.pdf]

Angewandte Chemie
Eine Zeitschrift der Gesellschaft Deutscher Chemiker

Supporting Information

Mechanistically Different Mechanochromophores Enable Calibration and Validation of Molecular Forces in Glassy Polymers and Elastomeric Networks

R. Hertel, M. Raisch, M. Walter, G. Reiter, M. Sommer**

Supporting Information for

Mechanistically Different Mechanochromophores Enable Calibration and Validation of Molecular Forces in Glassy Polymers and Elastomeric Networks

Raphael Hertel,*[a] Maximilian Raisch,[a] Michael Walter,[b] Günter Reiter[c] and Michael Sommer*[a]

[a] Institute for Chemistry, Chemnitz University of Technology, Chemnitz, Germany

[b] FIT Freiburg Center for Interactive Materials and Bioinspired Technologies, University of Freiburg, Freiburg, Germany

[c] Institute of Physics, Albert-Ludwig-University, Freiburg, Germany

Table of Contents

A General Materials and Methods 3

B Synthesis 5

Small Molecules 8

1′-ethyl-1′,3′-dihydro-3′,3′-dimethyl-5′,6-di(4-vinylphenyl)spiro[2*H*-1-benzopyran-2,2′-[2*H*]indole] (di(ethenylphenyl)-**SP**) 8

Polymer Synthesis 9

Suzuki polymerizations 9

Synthesis of α,ω-divinyl-P*mmp*P 9

Synthesis of **NO_2-SP**-P*mmp*P 11

Preparation of P*mmp*P films 11

Analysis of P*mmp*P polymers 12

Synthesis of PDMS samples 12

C Analysis of the mechanochromic response in P*mmp*P 13

Estimation of forces from the mechanochromic response of **DPP**-P*mmp*P and **TQxT**-P*mmp*P 13

Calculation of the force per chain from the macroscopic stress for P*mmp*P 14

Measurement of the mechanochromism of **SP**-P*mmp*P and **NO_2-SP**-P*mmp*P 15

D Analysis of mechanochromic response in PDMS 16

Mechanochromism of **DPP**-PDMS 16

Measurement of the mechanochromism of **SP**-PDMS and **NO_2-SP**-PDMS 17

E NMR spectra 19

F Supporting references 22

A General Materials and Methods

Chemicals. All chemicals, reagents and solvents were purchased from commercial sources and used as received unless otherwise noted. The solvents used for column chromatography were distilled under reduced pressure prior to use. Toluene was dried using Na and stored over molecular sieves 4 Å. Di(bromophenoxybutyl)-**DPP**,[1] dibutenyl-**DPP**,[1] dibromo-**TQxT**,[2] dibromo-**SP**,[3] dihydroxy-**NO_2-SP**,[4] divinyl-**NO_2-SP**[4] were prepared as previously reported. *p*-xylene and chlorobenzene were filtered over basic alumina.

NMR Spectroscopy. NMR spectra were recorded on an Avance NEO 600 FT spectrometer (^{1}H: 600 MHz, ^{13}C: 150.9 MHz). The spectra were referenced to the residual solvent peaks ($CHCl_3$: $\delta(^1H)$ = 7.26 ppm, $\delta(^{13}C)$ = 77.16 ppm. The analysis was executed with Mestrelab Mestrenova v11.0.4. Peak assignments are supported by HSQC and HMBC experiments.

High resolution mass spectra (HRMS) were obtained from a Bruker Trapped Ion Mobility Spectrometry (tims) time-of-flight (TOF) mass spectrometer in ESI+ mode in ethyl acetate solution.

Size Exclusion Chromatography (SEC). Molecular weights were measured on a Shimadzu system comprising a 5 µm precolumn and three SDplus columns with pore sizes ranging from 10^2 to 10^4 Å (Polymer Standards), connected in series with a RID20A RI detector and a FSPD-20AV photodiode array UV-vis detector (Shimadzu). Polystyrene standards were used for calibration. The samples were eluted with THF at 40 °C with a flow rate of 1.0 mL min^{-1}.

UV-vis Spectroscopy. All UV-vis spectra were measured in transmission. Thin-film absorption spectra were recorded on a Flame-S UV-vis spectrometer from Ocean Optics with an integration time of 3 ms and 666 scans to average, using OceanView 1.5.2 software. Thin-film absorption spectra were smoothed via Savitzky-Golay smoothing using third order polynomial considering 51 pts.

Photoluminescence (PL) Spectroscopy. PL spectra of thin films were recorded on a Flame-S UV-vis spectrometer (see above) with an integration time of 333 ms and 6 scans to average. For excitation during thin-film emission measurement a UV LED (Nichia NVSU233A UV SMD-LED, 365 nm, max. 1030 mW) operated with 900 mA at a distance of 10 cm was used. Emission spectra of thin films were calibrated using a relative intensity calibration obtained from a blackbody spectrum (2700 K) and smoothed via Savitzky-Golay smoothing using third order polynomial considering 21 pts.

Differential Scanning Calorimetry. DSC measurements were performed on a DSC 2500 (TA Instruments) in standard aluminum pans under a nitrogen atmosphere with a heating / cooling rate of 10 K min^{-1}. Sample masses were between 3.5 and 5.5 mg.

Tensile Testing. Stress-strain experiments were carried out on a Linkam TST-350 using a displacement ramp of 5 mm min^{-1}. A standard specimen shape according to DIN 53504 type 3 was used for all

poly(*meta*,*meta*,*para*-phenylene) (P*mmp*P) samples. Film thicknesses of the P*mmp*P samples were between 110 and 150 µm. PDMS samples were cut using a custom-made cutting die with a minimal width of 3.3 mm located in the center of the probes. PDMS films had a thickness of 260–330 µm. The samples were clamped with constant pressure using a torque tool. For both shapes, the engineering strain ε was calculated with $l_0 = 15$ mm. The shown values of stress are engineering stress, which were calculated by considering the respective constant cross-sectional area of the specimen at $\varepsilon = 0\%$.

B Synthesis

SP-P*mmp*P was prepared according to reported protocols by copolymerization.[5] The covalent incorporation of **NO_2-SP** into the P*mmp*P backbone was not straightforward, as **NO_2-SP** decomposed under basic conditions, especially using non-sterically hindered bases. This did neither allow for the functionalization with base-stable bromophenyl groups (**Scheme S1**) nor the incorporation into P*mmp*P using Suzuki polycondensation. Therefore, α,ω-divinyl-P*mmp*P oligomers were synthesized and further copolymerized with bisvinyl-functionalized **NO_2-SP** under acyclic diene metathesis (ADMET) conditions (**Scheme S3**). It is important to note that we did not observe any differences in the molecular, thermal, and mechanical properties of the resulting **NO_2-SP**-P*mmp*P prepared via ADMET compared to those of all other P*mmp*P samples used herein (**Figures 3** and **S1**).

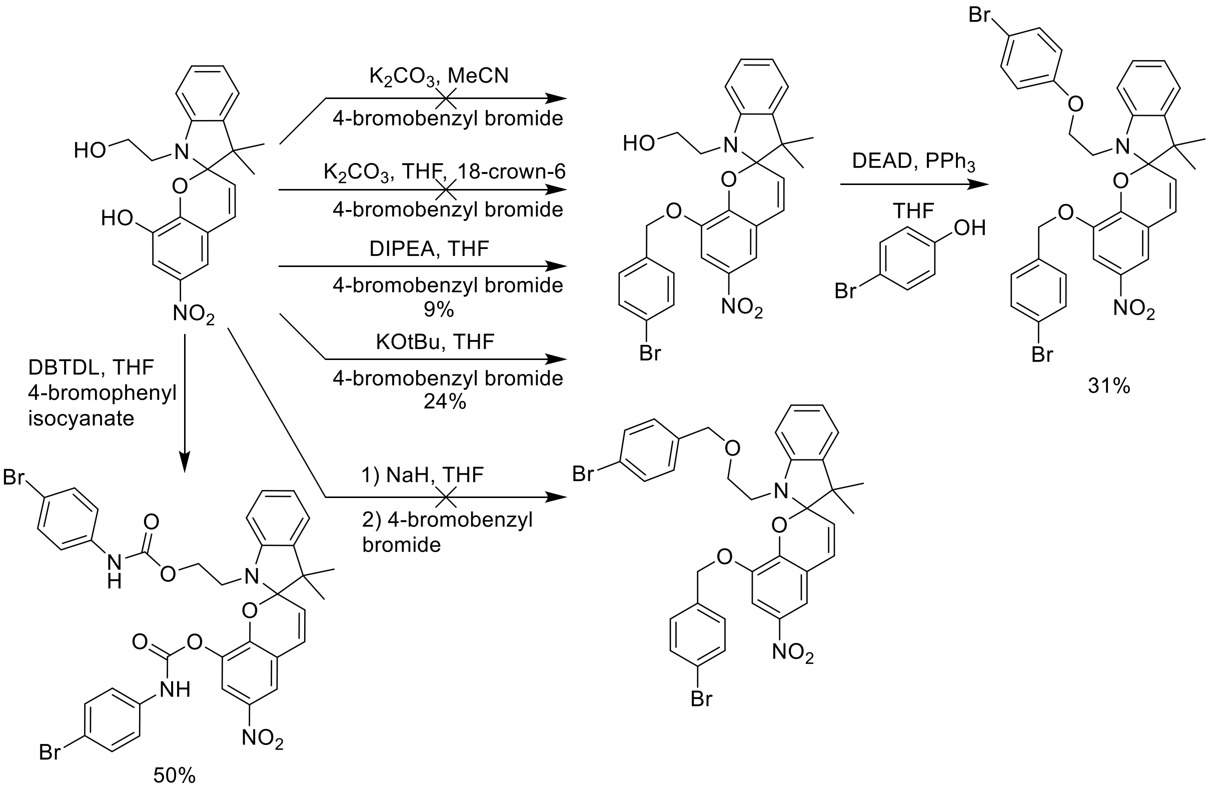

Scheme S1. Attempted unsuccessful and successful syntheses of di(bromophenyl)-substituted nitro-spiropyran

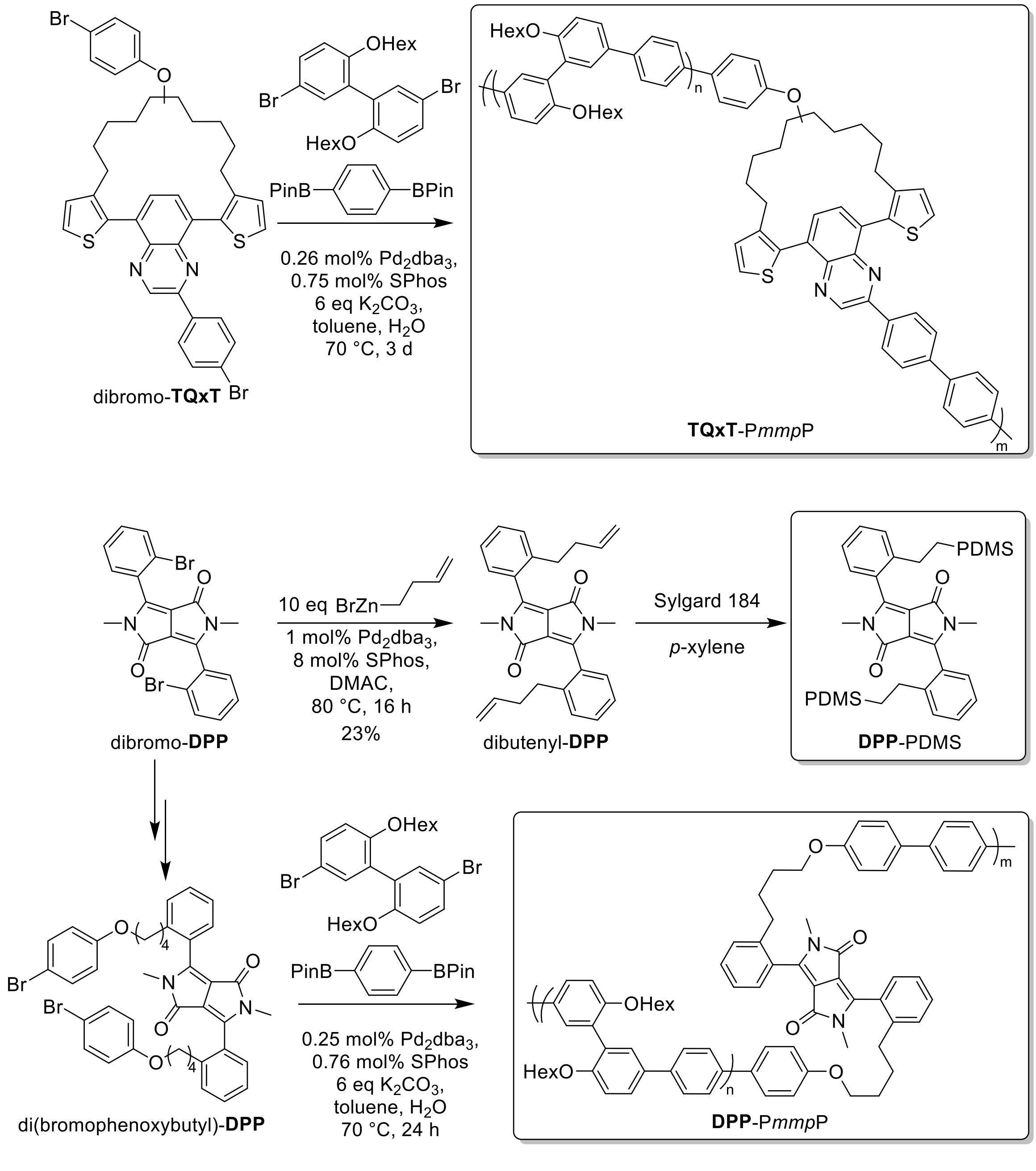

Scheme S2. Synthesis of the **TQxT**- and **DPP**-functionalized polymer materials.

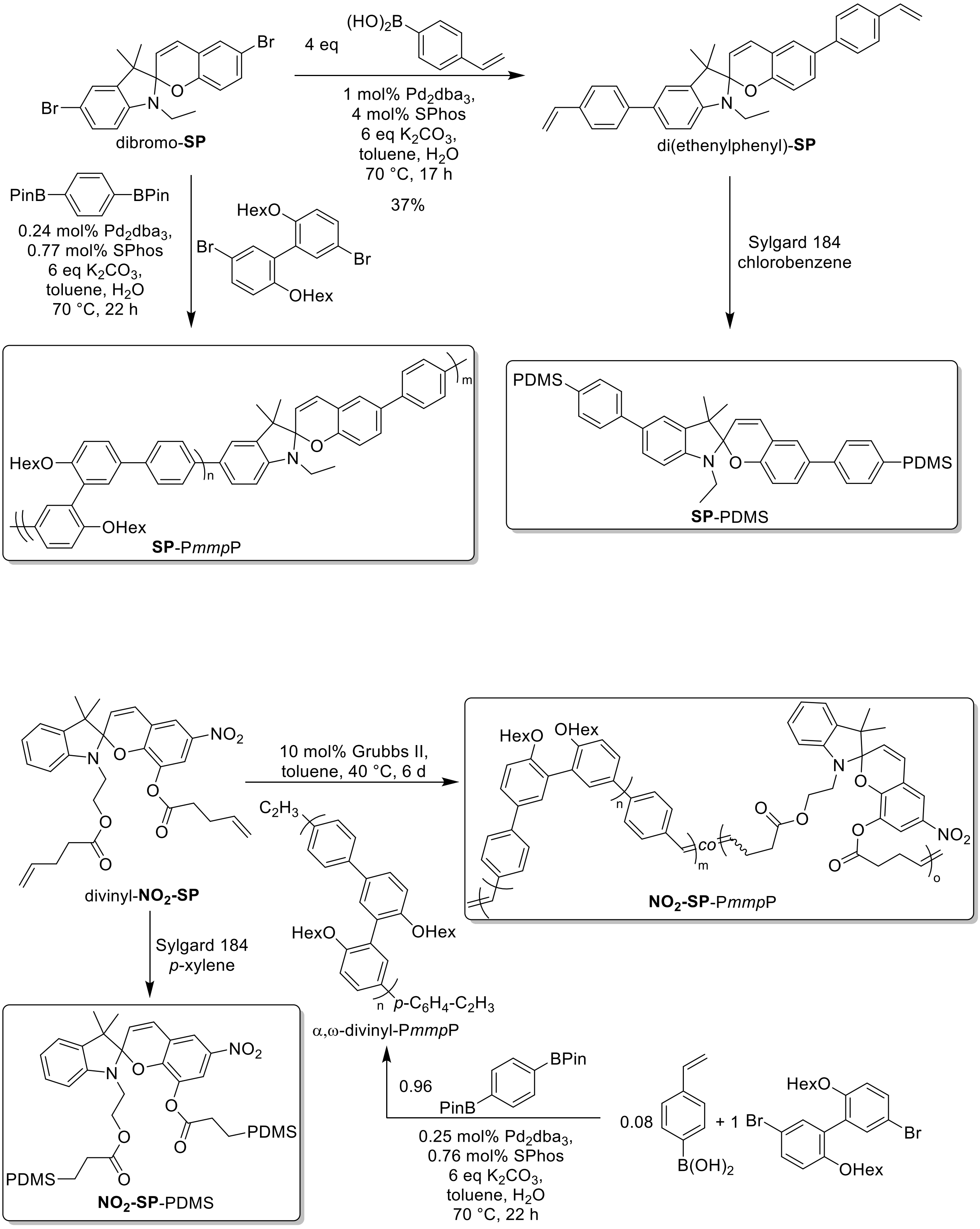

Scheme S3. Synthesis of the **SP**- and **NO2-SP**-functionalized polymers.

Small Molecules

1′-ethyl-1′,3′-dihydro-3′,3′-dimethyl-5′,6-di(4-vinylphenyl)spiro[2*H*-1-benzopyran-2,2′-[2*H*]indole] (di(ethenylphenyl)-SP)

A screw cap vial was charged with 1′-ethyl-1′,3′-dihydro-3′,3′-dimethyl-5′,6-dibromospiro[2*H*-1-benzopyran-2,2′-[2*H*]indole] (dibromo-**SP**) (0.10009 g, 0.223 mmol), 4-vinylbenzeneboronic acid (0.13182 g, 0.891 mmol, 4.00 eq), $Pd_2dba_3{\cdot}CHCl_3$ (0.00252 g, 0.002 mmol, 0.01 eq), SPhos (0.00393 g, 0.010 mmol, 0.04 eq) and K_2CO_3 (0.18499 g, 1.336 mmol, 6.00 eq).·After evacuating this mixture and backfilling with Ar four times, toluene (2.4 mL, degassed) and H_2O (2.8 mL) were added. The flask was sealed with PTFE tape and the reaction mixture was heated at 70 °C for 17 h under vigorous stirring. After that, toluene was removed under reduced pressure at room temperature. The resulting solid was filtrated from the aqueous phase, washed with water (2 mL) and extracted with a mixture of petroleum ether and ethyl acetate (v / v = 1 / 1, 3 × 3 mL). After removal of the solvents under reduced pressure the product di(ethenylphenyl)-**SP** (0.04060 g, 37% yield) was isolated by column chromatography using silica gel (prior deactivated with 1 vol% Et_3N) with a mixture of petroleum ether and ethyl acetate (v / v = 15 / 1, R_F = 0.41) as a colorless to pale yellow solid and could be crystallized from ethanol.

^{1}H NMR (600.1 MHz, $CDCl_3$) δ / ppm 7.55 (d, $^3J_{HH}$ = 8.0 Hz, 2 H, 2''-H), 7.51 (d, $^3J_{HH}$ = 8.0 Hz, 2 H, 2'''-H), 7.48–7.40 (m, 5 H, 6'-H, 3''-H, 3'''-H), 7.35 (dd, $^3J_{HH}$ = 8.4 Hz, $^4J_{HH}$ = 2.1 Hz, 1 H, 7-H), 7.32 (d, $^4J_{HH}$ = 1.8 Hz, 1 H, 4'-H), 7.29 (d, $^4J_{HH}$ = 2.1 Hz, 1 H, 5-H), 6.91 (d, $^3J_{HH}$ = 10.1 Hz, 1 H, 4-H), 6.82–6.71 (m, 3 H, 8-H, 5''-H, 5'''-H), 6.62 (d, $^3J_{HH}$ = 8.0 Hz, 1 H, 7'-H), 5.81–5.72 (m, 3 H, 3-H, 6''-H, 6'''-H), 5.26 (d, $^3J_{HHcis}$ = 10.8 Hz, 1 H, 6'''-H), 5.24 (d, $^3J_{HHcis}$ = 10.8 Hz, 1 H, 6''-H), 3.40 (dq, $^2J_{HH}$ = 14.7 Hz, $^3J_{HH}$ = 7.4 Hz, 1 H, 8'-H), 3.26 (dq, $^2J_{HH}$ = 14.6 Hz, $^3J_{HH}$ = 7.2 Hz, 1 H, 8'-H), 1.38 (s, 3 H, 11'-H), 1.24–1.19 (m, 6 H, 9'-H, 10'-H);

^{13}C{^{1}H} NMR (150.9 MHz, $CDCl_3$) δ / ppm 154.0 (C8a), 146.9 (C7a'), 141.5 (C1''), 140.2 (C1'''), 137.5 (C3a'), 136.8 (C5''), 136.6 (C5'''), 136.1 (C4'''), 135.5 (C4''), 132.9 (C6), 131.6 (C5'), 129.5 (C4), 128.4 (C7), 126.8 (C2'''/C3'''), 126.7 (C2''/C3''), 126.7 (C2'''/C3'''), 126.7 (C2''/C3''), 126.6 (C6'), 125.3 (C5), 120.7 (C4'), 120.2 (C3), 118.9 (C4a), 115.7 (C8), 113.7 (C6'''), 113.2 (C6''), 106.5 (C7'), 105.0 (C2), 52.5 (C3'), 38.1 (C8'), 26.4 (C10'), 20.3 (C11'), 14.6 (C9');

HRMS (ESI): *m/z* calcd for $C_{36}H_{33}NO^+$: 495.2562 M$^+$, found: 495.2557.

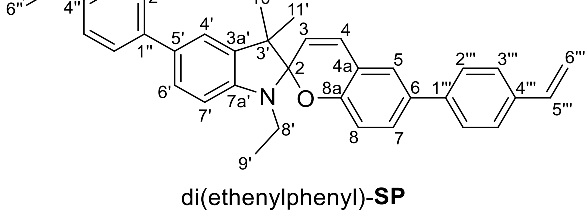

di(ethenylphenyl)-**SP**

Polymer Synthesis

Suzuki polymerizations

Pristine poly(*meta,meta,para*)phenylene (P*mmp*P) and spring-functionalized P*mmp*P (4 mol% **SP**-content: $M_{n,SEC}$ = 18 kg mol^{-1}, $M_{w,SEC}$ = 121 kg mol^{-1}; 0.06 mol% **DPP**-content: $M_{n,SEC}$ = 34 kg mol^{-1}, $M_{w,SEC}$ = 143 kg mol^{-1}; and 1.5 mol% **TQxT**-content: $M_{n,SEC}$ = 30 kg mol^{-1}, $M_{w,SEC}$ = 170 kg mol^{-1}) were synthesized according to the literature.[1,2,5]

Synthesis of α,ω-divinyl-P*mmp*P

To a Schlenk tube were added 1,4-benzenediboronic acid bis(pinacol)ester (1.23679 g, 3.747 mmol, 1 eq), 5,5'-dibromo-2,2'-di(hexoxy)biphenyl (2.0007 g, 3.905 mmol, 1.042 eq), 4-vinylbenzeneboronic acid (0.04622 g, 0.312 mmol, 0.083 eq), Pd_2dba_3·$CHCl_3$ (0.01007 g, 0.010 mmol, 0.003 eq), SPhos (0.01211 g, 0.029 mmol, 0.008 eq) and K_2CO_3 (3.23324 g, 23.395 mmol, 6.243 eq). 5 drops of Aliquat 336 were placed on the glass wall. The flask was evacuated and backfilled with Ar five times. Subsequently, toluene (10 mL, degassed) was added by syringe and the reaction mixture was mixed followed by the addition of water (12 mL, degassed). The biphasic mixture was vigorously stirred at 70 °C for 22 h. Then, 4-vinylbenzeneboronic acid (0.10485 g, 0.709 mmol, 0.189 eq), Pd_2dba_3·$CHCl_3$ (0.01005 g, 0.010 mmol, 0.003 eq) and SPhos (0.01226 g, 0.030 mmol, 0.008 eq) were transferred to the flask under Ar and the reaction continued at 70 °C. After 7 h, the reaction mixture was cooled to RT, the phases were separated after dilution with toluene (5 mL) and the polymer was precipitated from the organic phase into MeOH (300 mL). After filtration of the mixture, the solid was washed with MeOH (100 mL). The raw polymer was purified by Soxhlet extraction with *tert*-butyl methyl ether (TBME) and the product was collected with DCM yielding a colorless solid after drying in vacuum at 50 °C for 5 h (1.7058 g, quantitative yield, $M_{n,NMR}$ = 15.4 kg mol^{-1}, $M_{n,SEC}$ = 14.2 kg mol^{-1}, $M_{w,SEC}$ = 26.0 kg mol^{-1}).

^{1}H NMR (600.1 MHz, $CDCl_3$) δ / ppm 7.72−7.62 (m, 6 H, 2-H, 2'-H), 7.59 (d, 2 H, 6-H), 7.05 (d, 2 H, 5-H), 6.75 (dd, $^3J_{HHtrans}$ = 17.6, $^3J_{HHcis}$ = 10.9 Hz, –C*H*=CH$_2$), 5.77 (d, $^3J_{HHtrans}$ = 17.6, –CH=C*H*$_2$), 5.24 (d, $^3J_{HHcis}$ = 10.9 Hz, –CH=C*H*$_2$), 3.99 (t, 4 H, 7-H), 1.72−1.63 (m, 4 H, 8-H), 1.36−1.37 (m, 4 H, 9-H), 1.25−1.15 (m, 8 H, 10-H, 11-H), 0.85−0.76 (m, 6 H, 12-H).

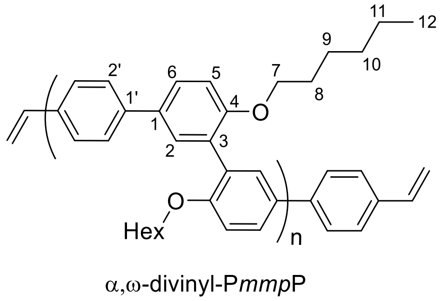

α,ω-divinyl-P*mmp*P

Table S1. Optimization of the reaction conditions for the synthesis of α,ω-divinyl-P*mmp*P

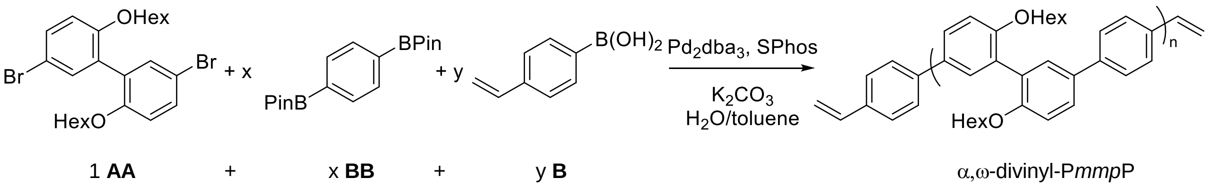

#	eq **BB**	eq **B** during polym.	eq **B** after polym.[a]	$M_{n,SEC}$ /kg mol^{-1}	$M_{w,SEC}$ /kg mol^{-1}	$Đ_{SEC}$	$M_{n,NMR}$[b] /kg mol^{-1}
1	0.980	-	0.510	26.2	121.9	4.7	195.8
2	0.960	-	0.670	23.4	96.8	4.1	97.1
2	0.940	0.060	0.362	16.3	31.2	1.9	16.8
3	0.960	0.080	-	12.4	24.5	1.9	17.2
4	0.960	0.080	0.181	14.2	26.0	1.8	15.4

[a] Added after 22 h polymerization time with fresh catalyst. [b] Determined from the ^{1}H NMR intensity ratio of the vinyl end group signals to the backbone signals.

Synthesis of NO_2-SP-P*mmp*P

A screw-cap vial charged with divinyl-**NO_2-SP** (17.44 mg, 0.033 mmol, 4.0 mol% based on the number of repeat units) and divinyl-terminated P*mmp*P (350.85 mg, $M_{n,NMR}$ = 15.4 kg mol^{-1}) was evacuated and backfilled with Ar four times. The mixture was dissolved in toluene (1.0 mL, dried and degassed) and a solution of Grubbs 2nd generation catalyst (2.96 mg, 0.003 mmol) in toluene (0.5 mL, dried and degassed) was added by syringe. The reaction mixture was stirred at 40 °C for 6 days periodically applying vacuum to remove ethene. Every second day, fresh Grubbs 2nd generation catalyst (2.22 mg, 0.003 mmol) in toluene (0.5 mL, dried and degassed) was added. After that, the viscous reaction mixture was diluted with toluene (1.5 mL). The polymer was precipitated in MeOH (150 mL), filtered off, washed with MeOH (3 × 20 mL) and purified by Soxhlet extraction with TBME. The product was collected by extraction with DCM yielding a brownish film. Remaining ruthenium was removed by mixing a solution of the polymer in toluene (4 mL) with aqueous 15% H_2O_2 solution (0.200 mL) for 15 minutes using a vortex mixer.[6] After dilution with toluene (1 mL), the polymer (335.95 mg, 91% yield, $M_{n,SEC}$ = 25.3 kg mol^{-1}, $M_{w,SEC}$ = 97.6 kg mol^{-1}) was collected by precipitation in MeOH (150 mL), washing with MeOH (3 × 20 mL), filtration and drying in high vacuum at 50°C for 5 h.

^{1}H NMR (600.1 MHz, $CDCl_3$) δ / ppm 7.93 (bs, 7''-H), 7.72−7.62 (m, 6 H, 2-H, 2'-H), 7.59 (d, 2 H, 6-H), 7.04 (d, 2 H, 5-H), 6.66 (bs, 7'''-H), 5.96 (bs, 3''-H), 4.12–3.85 (m, 4 H, –$CH_2(CH_2)_4CH_3$), 1.72−1.63 (m, 4 H, –$CH_2CH_2(CH_2)_3CH_3$), 1.36−1.37 (m, 4 H, –$(CH_2)_2CH_2(CH_2)_2CH_3$), 1.25−1.15 (m, 8 H, –$(CH_2)_3(CH_2)_2CH_3$), 0.85−0.76 (m, 6 H, –$(CH_2)_5CH_3$).

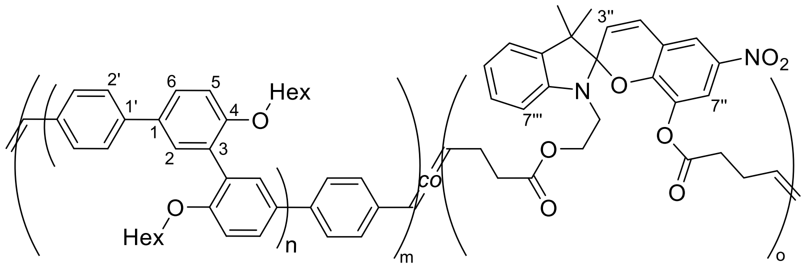

NO_2-SP-P*mmp*P

Preparation of P*mmp*P films

A DCM solution of the P*mmp*P polymer (2.00 mL, 100 mg mL^{-1}) was filtered through a pad of sea sand (1 cm, purified and calcinated) in a Petri dish (d = 35 mm). The solvent was allowed to evaporate at RT overnight with an inverted beaker covering the Petri dish. The resulting films had thicknesses between 110–150 µm. After the outer edge was removed, three dumbbell-shaped specimens were cut with a DIN 53504 type 3 cutting die per film.

Analysis of P*mmp*P polymers

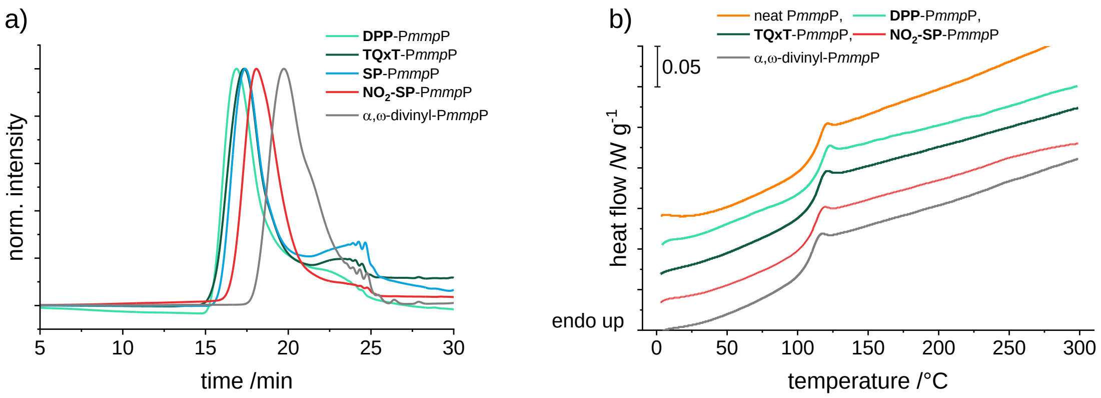

Figure S1. SEC chromatograms (a) and second heating scan of DSC analysis of the P*mmp*P samples.

Synthesis of PDMS samples

The preparation of **DPP** (6.32 mg, 0.015 mmol) in PDMS (2.5 mL Sylgard® 184 base) and **NO_2-SP** (18.91 mg, 0.036 mmol) in PDMS (2.5 mL Sylgard® 184 base) was carried out as previously reported.[1,4]

SP-PDMS was prepared as follows:

Di(ethenylphenyl)-**SP** (18.85 mg, 0.038 mmol) was dissolved in chlorobenzene (0.25 mL). The solution was mixed with Sylgard® 184 base (2.5 mL) in a 10 mL vial using a vortex mixer, and then curing agent (0.25 mL) was added. After vigorous mixing and removal of bubbles in vacuo, a film was doctor-bladed on surfactant-coated glass. Curing was conducted at 60 °C for 19 h in an oven. The film was peeled off and covered with weighing paper prior to cutting of the tensile testing specimens.

C Analysis of the mechanochromic response in P*mmp*P

Estimation of forces from the mechanochromic response of DPP-P*mmp*P and TQxT-P*mmp*P

The simulation of the force-dependent mechanochromic shift for the four most stable conformers of **DPP** as well as **TQxT** is detailed in previous publications, including the calculation of the proportionality factors between applied force and mechanochromic shifts.[1,2]

The shifts of the emission band maxima for both **DPP**-P*mmp*P as well as **TQxT**-P*mmp*P are monitored in dependence of the strain ε. The mechanochromic shift of the emission wavelength $\Delta\lambda_{em,DPP}$ from **DPP** is proportional to the applied forces below 1 nN in our simulations with a resulting conversion factor of 17 nm nN^{-1}.[1] For example, in a representative measurement $\Delta\lambda_{em,DPP}$ is 8.2 nm at $\varepsilon = 270\%$. Thus, we calculate a force of 0.48 nN at that ε. For **TQxT**-P*mmp*P, the irreversible mechanochromic part $\Delta\lambda_{em\ ring\ flip}$ = 16.8 nm resulting from the thiophene ring flip at forces > 27 pN needs to be subtracted from the overall mechanochromic shift $\Delta\lambda_{em,TQxT}$ to leave the mechanochromic shift resulting from reversible donor (D)-acceptor (A) planarization. The remaining reversible part of the mechanochromic shift is proportional to the applied forces below 1 nN in our simulations with a resulting conversion factor of 21 nm nN^{-1}.[2] In a representative measurement the overall mechanochromic shift of **TQxT**-P*mmp*P $\Delta\lambda_{em,TQxT}$ was 26.2 nm at $\varepsilon = 270\%$. Subtracting $\Delta\lambda_{em,ring\ flip}$ leaves 9.4 nm reversible mechanochromic shift and thus a force of 0.45 nN.

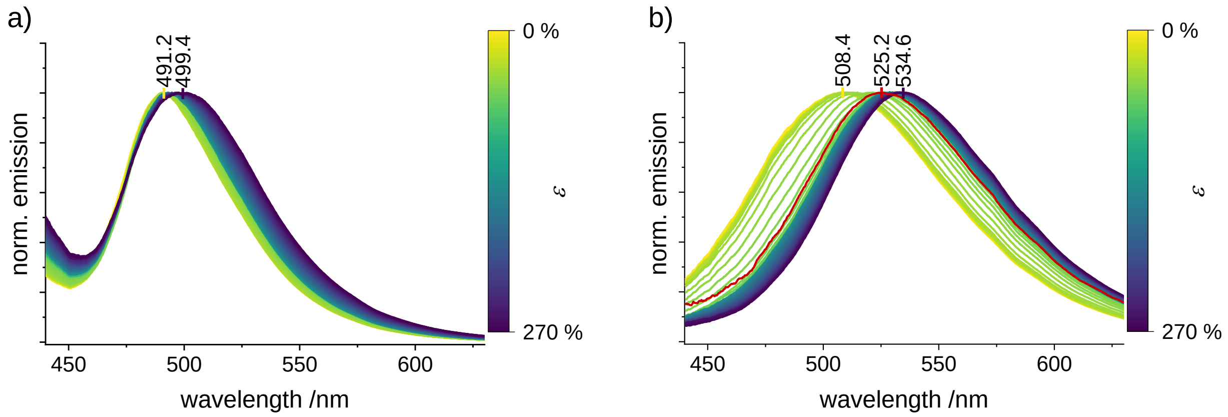

Figure S2. Representative change in normalized PL emission spectra of a) **DPP**-P*mmp*P and b) **TQxT**-P*mmp*P during straining and emission spectrum after sample failure (red line).

To analyse the accuracy of the linear correlation between mean macroscopic stress and determined mean molecular force for $\varepsilon > 50\%$ the mean molecular forces are depicted as function of the mean macroscopic stress in Figure S3.

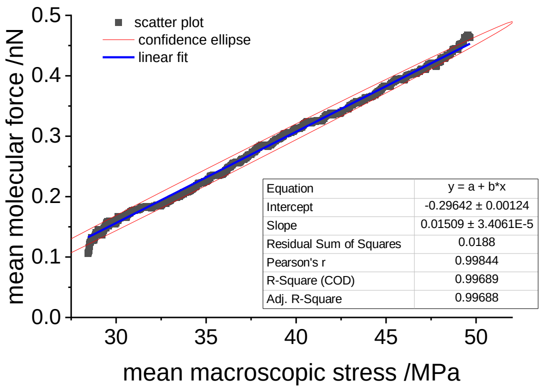

Figure S3. Linear fit of the determined mean molecular force and measured mean macroscopic stress in P*mmp*P for $\varepsilon > 50\%$.

Calculation of the force per chain from the macroscopic stress for P*mmp*P

To estimate the force per polymer chain, the macroscopic true stress can be multiplied by the cross-sectional area of one polymer chain A_{chain}. One approach to estimate $A_{\mathrm{chain}}$is given in equation S1[7]

$$A_{\mathrm{chain}} = \frac{m_{\mathrm{repeat\ unit}}}{\rho \cdot d} \tag{S1}$$

where $m_{\mathrm{repeat\ unit}}$ and d are the mass and the length of one repeat unit, respectively, and $\rho = 0.96\ \mathrm{g\ cm^{-3}}$[8] is the density of the material. Using 500 conformers created with RdKIT,[9] we obtain the expectation value d = 1.1 nm resulting in an estimated mean cross-sectional area of $\bar{A}_{\mathrm{chain}}$ = 0.7 nm^2.

The true stress at break obtained from the force at break divided through the cross-sectional area of the specimen after failure amounts to 220 N mm^{-2}. The force per chain approximated with $\bar{A}_{\mathrm{chain}}$ from equation S1 is 0.15 nN.

We additionally estimated the force per entanglement using the entanglement density ρ_{e} obtained from ρ and the entanglement molecular weight $M_{\mathrm{e}} = 4.8\ \mathrm{kg\ mol^{-1}}$[8]:

$$\rho_{\mathrm{e}} = \frac{\rho}{M_{\mathrm{e}}} \cdot N_{\mathrm{A}} = 1.20 \cdot 10^{26}\ \mathrm{m^{-3}} \tag{S2}$$

Under the assumption that the entanglement density is isotropic, the mean distance between entanglements d_{e} can be described as[10]:

$$d_{\mathrm{e}} = \rho_{\mathrm{e}}^{-\frac{1}{3}} \tag{S3}$$

Thus, the force per entanglement F_{e} results using the entanglement density per unit area and macroscopic true stress $\sigma = 220\ \mathrm{N\ mm^{-2}}$ using equation S4[11]

$$F_{\mathrm{e}} = \sigma \cdot d_{\mathrm{e}}^2 = 0.9\ \mathrm{nN} \tag{S4}$$

Measurement of the mechanochromism of SP-P*mmp*P and NO_2-SP-P*mmp*P

Representative UV-vis measurements for **SP**-P*mmp*P and **NO_2-SP**-P*mmp*P are shown in **Figure S3**. For analysis of the mechanochromic response of spiropyrans in P*mmp*P the resulting absorption band was integrated. A linear baseline was previously subtracted to remove the effect from light scattering at the aligned polymer chains after neck formation. Due to thinning of the film during straining, the regarding merocyanine absorption band intensities were divided through the matrix absorbance band integrals.

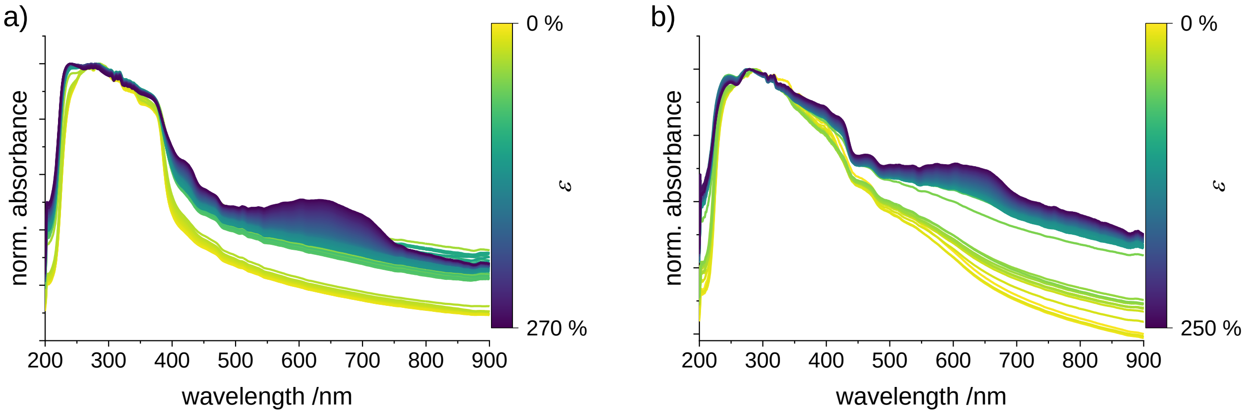

Figure S4. Representative normalized UV-vis spectra of a) **SP**-P*mmp*P and b) **NO_2-SP**-P*mmp*P during deformation.

The P*mmp*P matrix absorbs the UV part (< 350 nm) of the light used for the UV-vis measurements.[5] Thus, no difference in mechanochromism of **NO_2-SP**-P*mmp*P was observed when UV light was turned on or off during UV-vis measurements. Even when a film is irradiated with the deuterium lamp used for the UV-vis measurements for 20 min (twice the time for one stress-strain measurement), merocyanine formation was not observed (**Figure S4**).

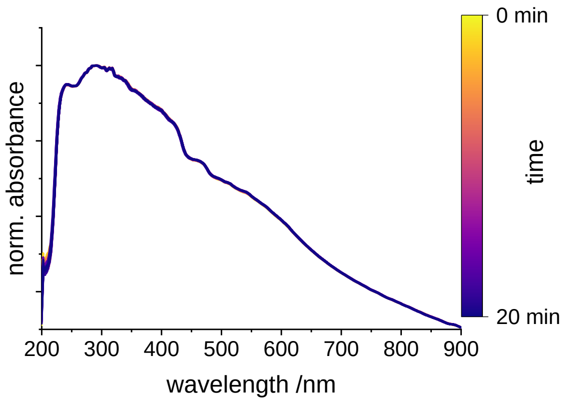

Figure S5. UV-vis spectra of **NO_2-SP**-P*mmp*P during irradiation with the deuterium lamp used for UV-vis measurement without straining.

D Analysis of mechanochromic response in PDMS

Mechanochromism of DPP-PDMS

For the PDMS samples, the specimens were cut using a custom-made double-bell-shaped cutting die (see inset of **Figure 5**). The calculation of molecular forces that occur in PDMS during tensile testing was performed in analogy to the procedure for P*mmp*P. The total mechanochromic shift $\Delta\lambda_{em,DPP}$ was again divided by the conversion factor of 17 nm nN^{-1}.[1] At $\varepsilon < 50\%$, small forces < 0.02 nN were detected due to slight buckling of the PDMS films after clamping.

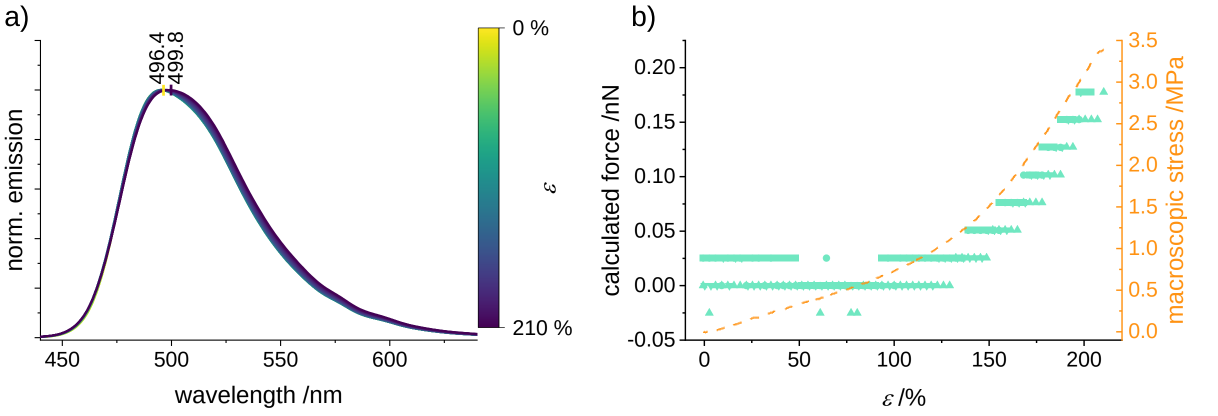

Figure S6. a) Representative normalized PL spectra of **DPP**-PDMS during straining. b) Molecular force obtained from the mechanochromic shift of the emission wavelengths and the theoretical force dependence of the CT wavelength for four tensile tests of **DPP**-PDMS (green symbols) and a representative stress-strain curve (dashed yellow line).

The accuracy of the linear correlation between mean macroscopic stress and determined mean molecular force in PDMS was determined for $\varepsilon > 120\%$ (onset of the mechanochromic response) in Figure S7.

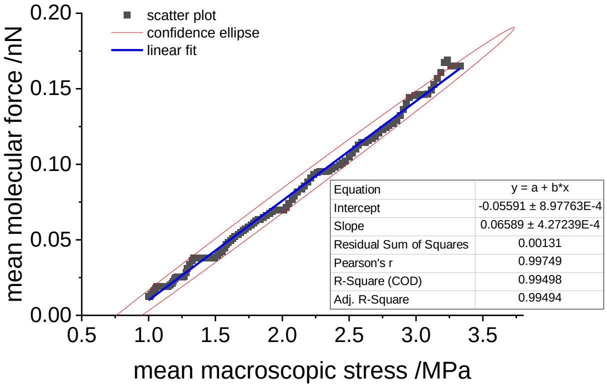

Figure S7. Linear fit of determined mean molecular force and measured mean macroscopic stress in PDMS for $\varepsilon > 120\%$.

Measurement of the mechanochromism of SP-PDMS and NO_2-SP-PDMS

The UV-vis spectra for **NO_2-SP**-PDMS were recorded with the deuterium lamp turned off. For **SP**-PDMS the absorbance spectra were measured with the deuterium lamp switched on, which did not lead to light-induced **SP** activation. Representative UV-vis spectra during deformation of the respective merocyanine forms **MC**-PDMS and **NO_2-MC**-PDMS are shown in **Figure S6**.

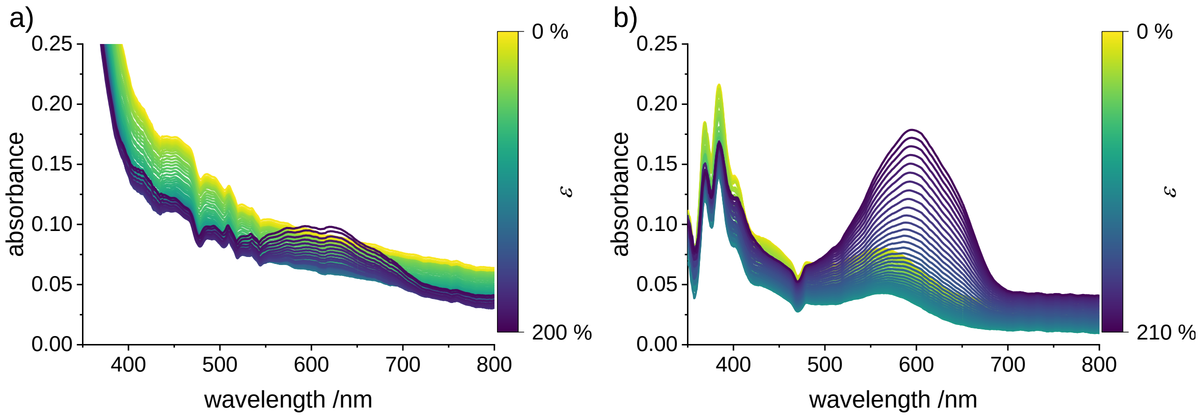

Figure S8. Representative UV-vis spectra of **MC**-PDMS (a) and **NO_2-MC**-PDMS (b) during straining.

The absorbances of **MC**-PDMS and **NO_2-MC**-PDMS relative to the matrix absorbance in dependence of ε are compared in **Figure S7** for multiple measurements.

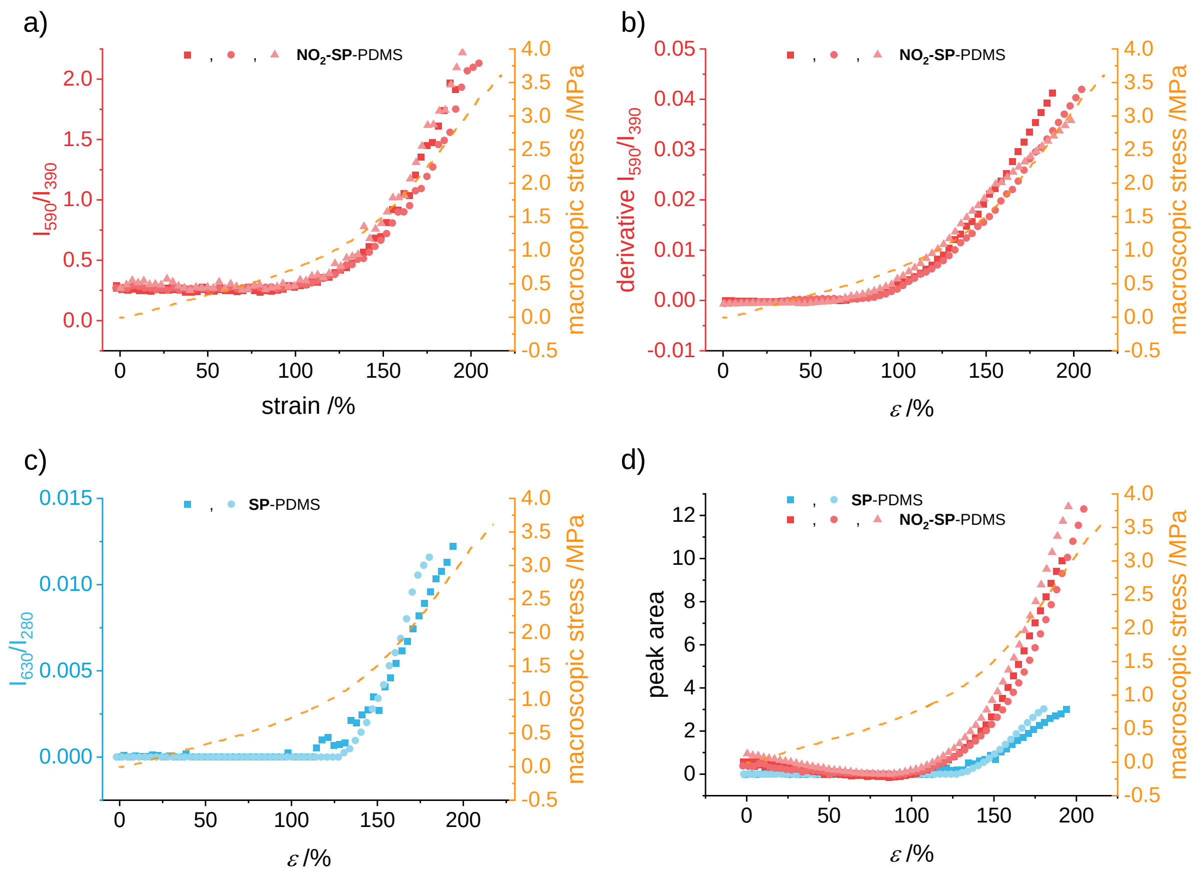

Figure S9. Relative UV-vis absorbance of **NO_2-MC**-PDMS (a) and **MC**-PDMS (c) as well as the first derivative of the relative UV-vis absorbance of **NO_2-MC**-PDMS (b) in dependence of ε in PDMS. d) Comparison of the merocyanine UV-vis peak area of **MC**-PDMS and **NO_2-MC**-PDMS. Representative stress-strain curves are shown as dashed yellow line.

E NMR spectra

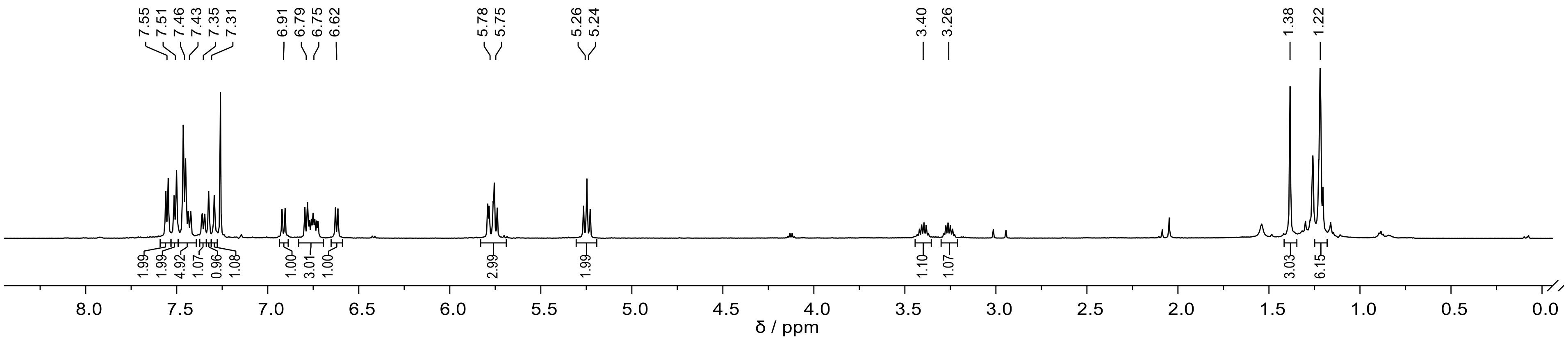

Figure S10. ^{1}H NMR spectrum of di(ethenylphenyl)-**SP** in $CDCl_3$.

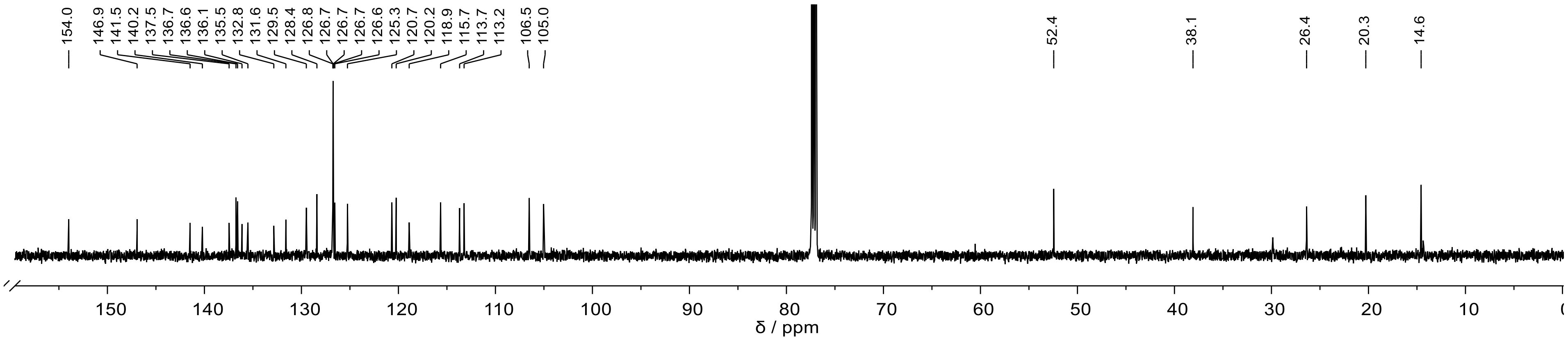

Figure S11. $^{13}C\{^{1}H\}$ NMR spectrum of di(ethenylphenyl)-**SP** in $CDCl_3$.

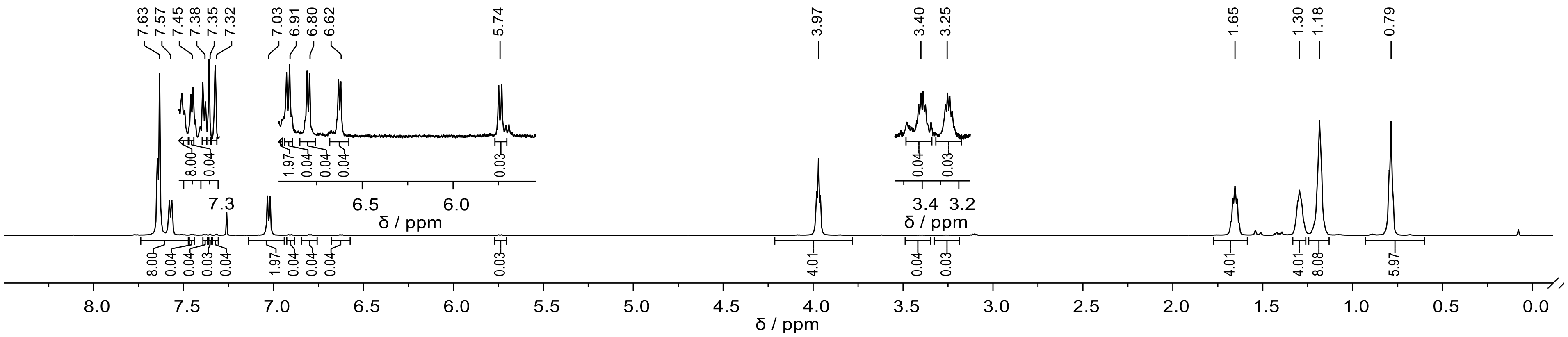

Figure S12. ^{1}H NMR spectrum of **SP**-P*mmp*P in $CDCl_3$.

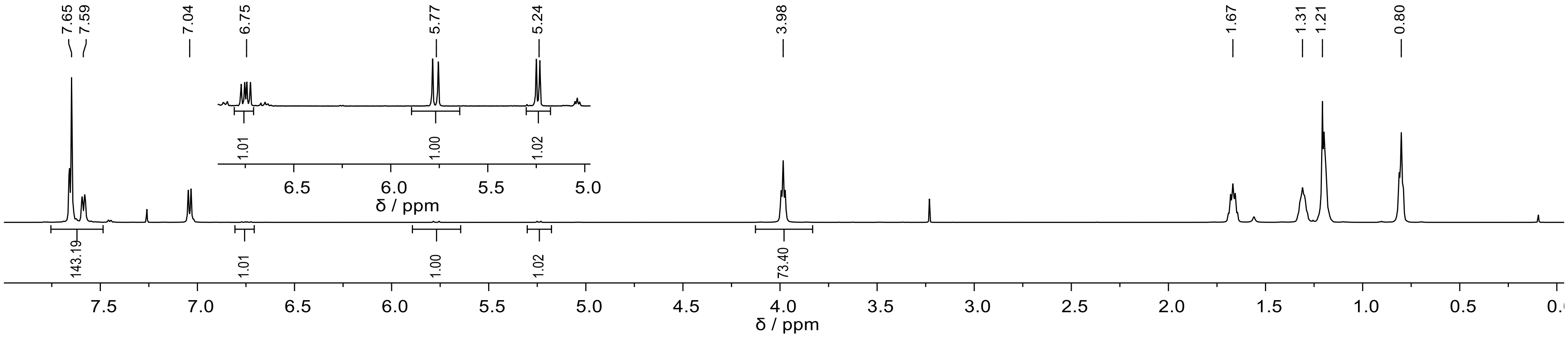

Figure S13. ^{1}H NMR spectrum of α,ω-divinyl-P*mmp*P in $CDCl_3$.

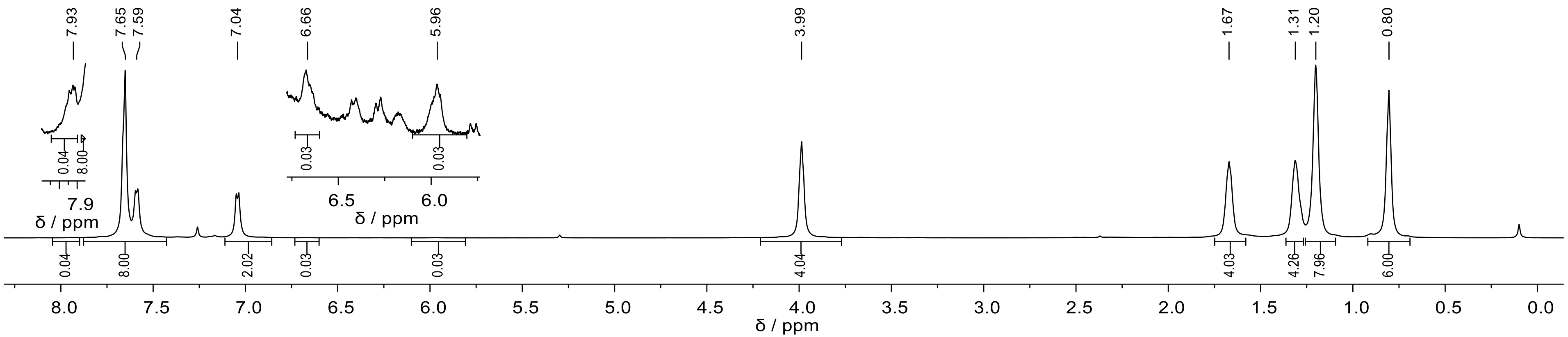

Figure S14. ^{1}H NMR spectrum of **NO_2-SP**-P*mmp*P in $CDCl_3$.

F Supporting references

[1] M. Raisch, W. Maftuhin, M. Walter, M. Sommer, *Nat. Commun.* **2021**, *12*, 4243.
[2] R. Hertel, W. Maftuhin, M. Walter, M. Sommer, *J. Am. Chem. Soc.* **2022**, *144*, 21897–21907.
[3] M. Sommer, H. Komber, *Macromol. Rapid Commun.* **2013**, *34*, 57–62.
[4] G. R. Gossweiler, G. B. Hewage, G. Soriano, Q. Wang, G. W. Welshofer, X. Zhao, S. L. Craig, *ACS Macro Lett.* **2014**, *3*, 216–219.
[5] F. Kempe, O. Brügner, H. Buchheit, S. N. Momm, F. Riehle, S. Hameury, M. Walter, M. Sommer, *Angew. Chem.* **2018**, *130*, 1009–1012.
[6] D. W. Knight, I. R. Morgan, A. J. Proctor, *Tetrahedron Lett.* **2010**, *51*, 638–640.
[7] P. I. Vincent, *Polymer* **1972**, *13*, 558–560.
[8] A. M. Fenton, R. Xie, M. P. Aplan, Y. Lee, M. G. Gill, R. Fair, F. Kempe, M. Sommer, C. R. Snyder, E. D. Gomez, R. H. Colby, *ACS Cent. Sci.* **2022**, *8*, 268–274.
[9] S. Riniker, G. A. Landrum, *J. Chem. Inf. Model.* **2015**, *55*, 2562–2574.
[10] T. Goda, J. Watanabe, M. Takai, K. Ishihara, *Polymer* **2006**, *47*, 1390–1396.
[11] K. Zheng, Y. Zhang, B. Li, S. Granick, *Nat. Commun.* **2023**, *14*, 537.
